# Classical swine fever virus recruits ALIX and ESCRT-III to facilitate viral budding

**DOI:** 10.1128/mbio.02618-24

**Published:** 2025-02-25

**Authors:** Jinxia Chen, Hanfei Yang, Mingyue Wan, Yan Cheng, Jishan Bai, Yuhang Li, Jing Chen, Bingqian Zhao, Fei Gao, Bin Zhou

**Affiliations:** 1 MOE Joint International Research Laboratory of Animal Health and Food Safety, College of Veterinary Medicine, Nanjing Agricultural University, Nanjing, China; 2 Shanghai Veterinary Research Institute, Chinese Academy of Agricultural Sciences 118161 , Shanghai, China; The University of North Carolina at Chapel Hill, Chapel Hill, North Carolina, USA; Cincinnati Children's Hospital Medical Center, Cincinnati, Ohio, USA

**Keywords:** CSFV, budding, ESCRT, ALIX, vesicle transport

## Abstract

Classical swine fever virus (CSFV) incurs substantial economic losses in the global swine industry due to its persistent emergence and re-emergence across various countries. However, the precise mechanisms governing CSFV budding remain inadequately understood. Our study elucidates that the endosomal sorting complex required for transport (ESCRT)-associated protein ALIX, in conjunction with ESCRT-III, plays a pivotal role in orchestrating CSFV budding. Genomic sequence analysis identified a critical interaction between the YPXnL late domain on the E2 protein and ALIX. Through immunoprecipitation and structural domain deletion assays, we demonstrated that the ALIX Bro1 domain specifically recognized viral particles by binding to the YPXnL motif. Immunoelectron and transmission electron microscopy further confirmed that, upon infection, ALIX accumulated at the periphery of subcellular organelles, including COPII vesicles, endosomes, and the Golgi apparatus, thereby facilitating CSFV budding. Our findings also revealed that ESCRT-III subunits CHMP2B, CHMP4B, CHMP7, and VPS4A interacted with ALIX to expedite CSFV budding. Notably, Rab8 activated by Kif4A contributed to the release of CSFV particles by interacting with ALIX and directing ALIX-containing vesicles along microtubules toward the cytosol. Our study demonstrates that ALIX specifically recognizes E2 and orchestrates the recruitment of ESCRT-III and Rab8 to facilitate the vesicular budding of CSFV particles from the Golgi apparatus to the cytosol. Ultimately, virus-laden vesicles propelled by Kif4A are transported along microtubules to the plasma membrane for release. Our findings offer the first comprehensive elucidation of the CSFV budding process and contribute to the identification of antiviral targets, thereby advancing the development of antiviral therapeutics.

**IMPORTANCE:**

The endosomal sorting complex required for transport (ESCRT) machinery plays a pivotal role in the sorting of membrane proteins in eukaryotic cells and regulating various stages of infection for numerous viruses. Previous studies have underscored the indispensable role of ESCRT in the cellular entry and replication of classical swine fever virus (CSFV). However, the precise mechanisms by which ESCRT recognizes CSFV particles and initiates viral vesicle budding have remained elusive. This study reveals that the Bro1 domain of ALIX initiates viral budding proximal to the Golgi apparatus by specifically recognizing the YPXnL late domain on the CSFV E2 protein. Mechanistically, ALIX and ESCRT-III facilitate Rab8-regulated endosomal transport of CSFV particles from the Golgi apparatus to the plasma membrane. Subsequently, virions are propelled by the kinesin Kif4A along microtubules for egress into the extracellular space. In summary, these findings significantly advance our understanding of CSFV pathogenesis and offer valuable insights into the vesicular transport and budding mechanisms of CSFV particles.

## INTRODUCTION

The endosomal sorting complex required for transport (ESCRT) is an evolutionarily conserved cellular machinery involved in a wide array of membrane-remodeling processes (1). Its core functions include the recognition, sorting, and trafficking of ubiquitinated membrane proteins into multivesicular bodies destined for lysosomal degradation (2, 3). Beyond these roles, ESCRT is indispensable for cytokinesis, plasma membrane repair, and nuclear envelope reassembly during mitosis. Prior research has established that ESCRT is crucial for orchestrating various stages of the viral life cycle, encompassing viral entry, replication, and budding within the host cell (4, 5). For instance, human cytomegalovirus recruits the essential ESCRT-III subunit VPS4A to the viral assembly complex to facilitate virion assembly. Similarly, the ESCRT-III subunit CHMP4C preserves the integrity of the endocytic pathway during herpes simplex virus 1 (HSV-1) infection, while TSG101 plays a pivotal role in regulating HSV-1/2 egress (6, 7). During HIV budding, ESCRT-III subunits, notably CHMP4B and VPS4A, assemble into a helical structure that facilitates the detachment of budding viral particles from the host cell membrane through a process of polymerization and contraction (8–10). Despite these advances, the intricate mechanisms by which ESCRT governs viral invasion, replication, assembly, and release, especially in emerging viruses that infect swine, remain incompletely understood. Further research is imperative to uncover novel antiviral targets and advance therapeutic strategies.

Classical swine fever (CSF) caused by the classical swine fever virus (CSFV) is a severe hemorrhagic disease in pigs manifesting with respiratory and gastrointestinal syndromes. It inflicts considerable economic damage globally and designated as a notifiable disease by the World Organization for Animal Health (11, 12). CSFV is classified within the *Pestivirus* genus of the *Flaviviridae* family. Its genome is a single-stranded, positive-sense RNA that is approximately 12.3 kb in length and encoding a polyprotein that comprises the 5′-UTR, an ORF, and the 3′-UTR. This polyprotein undergoes proteolytic processing to produce four structural proteins (capsid protein C, envelope glycoproteins Erns, E1, and E2) and eight non-structural proteins (Npro, p7, NS2, NS3, NS4A, NS4B, NS5A, and NS5B) (13–15). E2 is the principal immunogen of flaviviruses that is extensively utilized in their diagnosis and vaccine development (16, 17). Our previous studies have demonstrated that host cell proteins interact with CSFV non-structural proteins, playing an essential role in viral replication. CSFV enters host cells through clathrin- or caveolin-1-mediated endocytosis, a process that is cell type-dependent and requires cholesterol and low pH for efficient infection (18, 19). Members of the Rab protein family, including Rab5, Rab7, Rab11, and Rab18, are vital in mediating viral endocytosis (20, 21). Moreover, our recent studies have shown that CSFV exploits ESCRT components to facilitate its entry and replication processes (22, 23). A comprehensive investigation of the CSFV life cycle is imperative for the development of novel and effective antiviral agents and vaccines to mitigate CSF outbreaks. Nevertheless, the precise mechanisms governing CSFV particle budding remain insufficiently understood (24).

ESCRT proteins are integral to the infection cycle of various members of the *Flaviviridae* family. Studies suggest that while the key ESCRT subunits ALIX and VPS4 are not essential for the replication of dengue virus (DENV) and Japanese encephalitis virus (JEV), TSG101, CHMP2/3, and CHMP4 are crucial for their transmission (25, 26). The NS3 protein of yellow fever virus (YFV) interacts with ALIX, and the deletion of the YPTI motif in NS3 markedly impairs YFV egress (27). Although ESCRT is known to influence viral budding, the precise pathway of YFV translocation remains elusive. Our previous study demonstrated that nine ESCRT complex subunits (HRS/Tsg101/VPS28/VPS25/CHMP2B/CHMP4B/CHMP7/VPS4A and ALIX) induce the formation of ball-shaped intronic traps on the ER membrane by interacting with several CSFV nonstructural proteins (NS3, NS4B, NS5A, or NS5B). These interactions facilitate the assembly of the viral replication complex (VRC), with lipid droplets serving as an energy source for CSFV replication (22, 28). Interestingly, CSFV infection promoted the recruitment of four ESCRT subunits (CHMP2B, CHMP4B, CHMP7, and VPS4A) to form a complex that anchors the VRC at the ER membrane. This study delved into the novel role of the ESCRT complex in CSFV budding. We elucidated the molecular mechanism through which ESCRT orchestrated the budding of CSFV particles and uncovered that ESCRT plays a pivotal role in the transport of CSFV-laden vesicles from the Golgi apparatus to the plasma membrane, a process meticulously regulated by Rab8 along microtubules. Our findings advance the understanding of the CSFV budding stage, offering valuable insights for the identification of antiviral targets and the development of effective therapeutics.

## RESULTS

### ALIX is involved in CSFV budding

**Fig 1:**
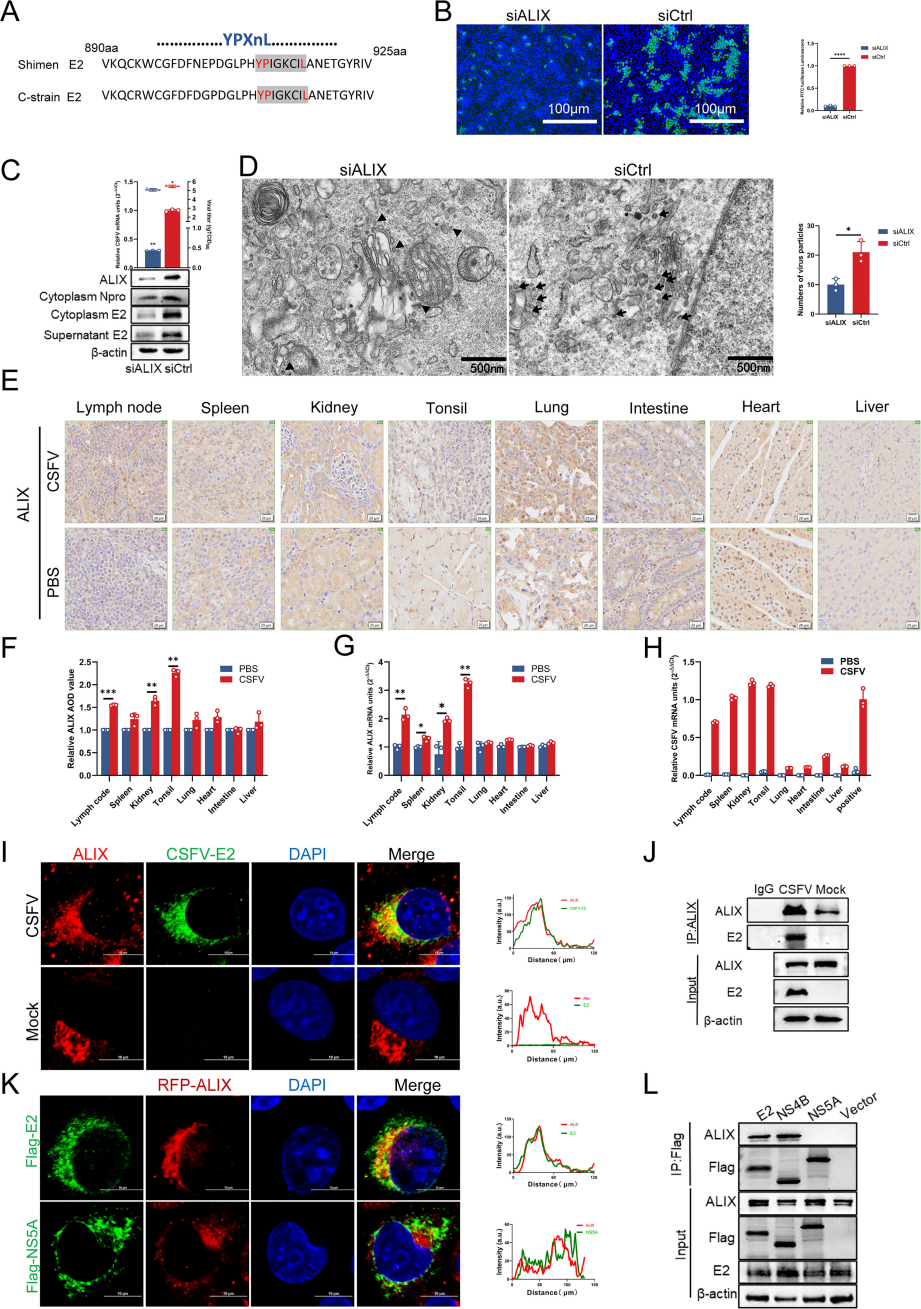
ALIX involvement in CSFV budding. (**A**) Sequence comparison of the CSFV Shimen and C strain genomes revealed the presence of the YPXnL late domain in the CSFV E2 coding regions (890–925aa). (**B**) PK-15 cells transfected with siALIX or siCtrl were inoculated with CSFV (MOI = 0.5) for 36 hpi. Cell supernatants containing progeny virus were collected to inoculate fresh cells. Cells were fixed with 4% PFA and subjected to indirect immunofluorescence experiment using the mouse anti-E2 monoclonal antibody (WH303, green). Nuclei were stained with 4′,6-diamidino-2-phenylindole (DAPI). (**C**) The reinfected cell cultures and supernatants were harvested and subjected to RT-qPCR, western blotting, and virus titration. (**D**) The infected cells transfected with siALIX or siCtrl were fixed and subjected to transmission electron microscopy. Viral particles around the Golgi apparatus were counted. (**E–H**) ALIX protein expression levels in the lymph nodes, spleen, kidney, lung, tonsil, heart, intestine, and liver of infected and uninfected pigs were measured through immunohistochemical assay (IHC). 16× magnification (scale bar, 20 µm). ALIX and CSFV mRNA levels in these organs above were measured using RT-qPCR. (**I**) PK-15 cells infected with CSFV for 36 h were fixed and subjected to confocal microscopy using rabbit anti-ALIX (red) and mouse anti-E2 antibodies (green). Nuclei were stained with DAPI (green). Nuclei were stained with DAPI. Bars = 10 µm. (**J**) Infected cells were harvested for immunoprecipitation using a rabbit anti-ALIX antibody. Whole-cell lysates were harvested and subjected to western blotting using mouse anti-E2 and rabbit anti-ALIX antibodies, with β-actin as a loading control. (**K**) PK-15 cells were transfected with plasmids (pFlag-E2, pFlag-NS5A, or pRFP-ALIX) for 24 h posttransfection (hpt), then infected with CSFV (MOI = 0.5) for 36 h. Cells were fixed and subjected to confocal microscopy using rabbit anti-ALIX (red) and mouse anti-Flag antibodies (green). Nuclei were stained with DAPI. Bars = 10 µm. (**L**) HEK-293T cells were transfected with plasmids (pFlag-E2, pFlag-NS4B, and pRFP-ALIX) for 36 h, then infected with CSFV (MOI = 0.5) for 36 h. Harvested cell cultures were subjected to western blotting using rabbit anti-Cherry and mouse anti-Flag antibodies, with β-actin as a loading control. Data are presented as the mean ± SD of data from three independent experiments. *P < 0.05, **P < 0.01, and ***P < 0.001.

ALIX, a pivotal auxiliary factor within the ESCRT system, interacts with the HIV Gag protein and is indispensable for HIV budding (29). Helical assemblies involving ALIX and ESCRT subunits are essential for retroviral budding (30). The late domain (YPXnL or PPXY), a specific motif in retroviruses, such as HIV, facilitates viral budding by engaging the ESCRT machinery of the host cell (31, 32). Although the critical role of ALIX in retrovirus budding is well established, its function in flavivirus budding remains poorly understood. To elucidate the role of ALIX in CSFV budding, we analyzed the gene sequences of the Shimen and C strains of CSFV. The genes encoding the envelope glycoprotein E2 contain YPXnL motifs, which are capable of binding to ALIX (Fig. 1A) (33). Additionally, we examined the genomes of other Pestivirus members, including additional CSFV and BDV strains, and identified YPXnL motifs in both (Fig. S1A; Table S1). To confirm the involvement of ALIX in CSFV budding, PK-15 cells were transfected with ALIX-targeting small interfering RNA (siALIX) or siCtrl prior to infection with CSFV (MOI = 0.5). At 36 h postinfection (hpi) at 37°C, cell supernatants were collected and subjected to infect fresh cells. The reinfected cells were then fixed and stained with mouse anti-E2 antibody for confocal microscopy. The results showed that ALIX knockdown significantly diminished fluorescence intensity, indicating reduced infection (Fig. 1B). Cell lysates and supernatants were collected 36 hpi and analyzed via RT-qPCR, western blotting, and viral titration. As depicted in Fig. 1C, the expression levels of CSFV Npro and E2 were markedly reduced in siALIX-treated cells compared to siCtrl-treated cells, underscoring the significant influence of ALIX on CSFV budding. RT-qPCR and viral titration assays further confirmed that viral mRNA levels and loads were significantly diminished in siALIX-treated cells. The effects of ALIX at different time points post-CSFV infection revealed that ALIX knockdown had a more pronounced inhibitory effect on the virus at 36 hpi, thereby further confirming the accuracy of our experimental conditions (Fig. S1B). Transmission electron microscopy (TEM) revealed a pronounced reduction in virus particles proximal to the Golgi apparatus in siALIX-treated cells (Fig. 1D). These *in vitro* data underscore the crucial role of ALIX in CSFV budding. To further investigate the relationship between ALIX expression and viral infection, we conducted *in vivo* challenge experiments and performed an immunohistochemical analysis on infected tissues. Comparative grayscale-value analysis across various tissues revealed that ALIX expression was significantly elevated in tissues with high viral loads compared to those with low viral loads and uninfected controls (Fig. 1E and F). The ALIX and CSFV mRNA levels in infected and PBS-treated pigs were measured using RT-qPCR. These results indicated that ALIX production was higher in tissues with high viral tropism (Fig. 1G and H). In summary, these *in vitro* and *in vivo* findings underscore the pivotal role of ALIX in CSFV budding.

To elucidate the interaction between ALIX and CSFV E2, infected cells were analyzed using confocal microscopy and co-immunoprecipitation (Co-IP) assays. The results revealed that endogenous ALIX associated with E2 upon infection (Fig. 1I and J). To further corroborate this interaction, cells were co-transfected with pFlag-E2 or pFlag-NS5A (negative control) and pRFP-ALIX, followed by confocal microscopy. The findings demonstrated a significant co-localization between RFP-ALIX and Flag-E2, whereas no co-localization was detected between RFP-ALIX and Flag- NS5A (Fig. 1K). Moreover, cells transfected with pRFP-ALIX and pFlag-E2, pFlag-NS5A (negative control), pFlag-NS4B (positive control) (22), or vector were subjected to Co-IP assay. Western blotting assay subsequently confirmed that ALIX specifically interacts with E2 (Fig. 1L). These results provide compelling preliminary evidence that ALIX modulates CSFV budding through its interaction with E2.

### ALIX Bro1 interacts with YPXnL motif of CSFV E2

**Fig 2:**
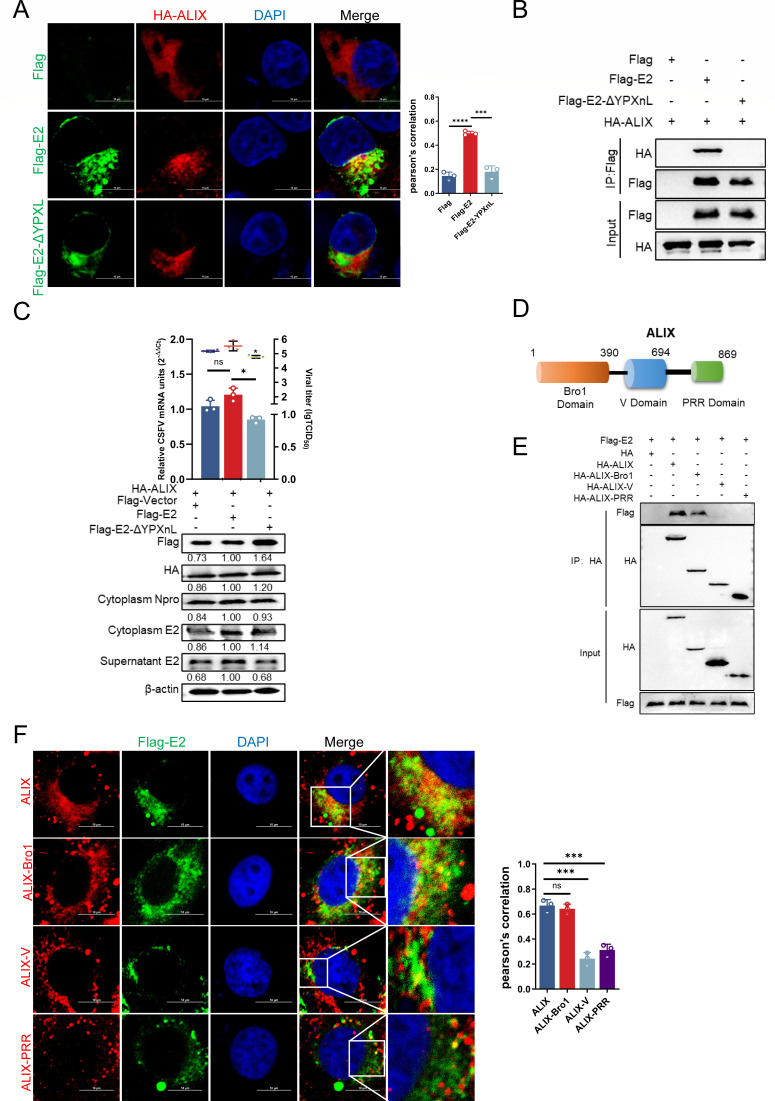
ALIX Bro1 interacts with the E2 YPXnL motif. (**A**) PK-15 cells co-transfected with the plasmids pHA-ALIX and pFlag vector, pFlag-E2, or pFlag-E2-ΔYPXnL for 24 h were fixed and subjected to confocal microscopy using rabbit anti-ALIX (red) or mouse anti-Flag antibody (green). Nuclei were stained with 4′,6-diamidino-2-phenylindole (DAPI). Scale bars = 10 µm. (**B**) HEK-293T cells were transfected with the plasmids above for 24 h, then harvested for immunoprecipitation using a mouse anti-Flag antibody. Whole-cell lysates were harvested and subjected to western blotting using rabbit anti-HA and mouse anti-Flag antibodies. (**C**) PK-15 cells were transfected with the plasmids above for 24 h and infected with CSFV (MOI = 0.5) for 36 h. The cell supernatants after reinfection were collected, and the virus loads were measured using titer titration (TCID50/mL). Cell cultures were harvested and subjected to RT-qPCR using the special primers and western blotting using mouse anti-E2 and rabbit anti-HA and mouse anti-Flag antibodies, along with β-actin as a loading control. (**D**) ALIX consists of three structural domains: the Bro1 (1–390aa), V (391–694aa), and PRR domains (695–869aa). (**E**) PK-15 cells were co-transfected with the plasmids pFlag-E2 and pHA-ALIX, pHA-ALIX-Bro1, pHA-ALIX-V, or pHA-ALIX-PRR for 24 h, then harvested for immunoprecipitation using a rabbit anti-HA antibody. Whole-cell lysates were harvested and subjected to western blotting using the rabbit anti-HA or mouse anti-Flag antibody. (**F**) PK-15 cells were co-transfected with the plasmids pFlag-E2 and pHA-ALIX, pHA-ALIX-Bro1, pHA-ALIX-V, or pHA-ALIX-PRR for 24 h and infected CSFV (MOI = 0.5) for 36 h. Cells were fixed and subjected to confocal microscopy using rabbit anti-ALIX (red) and mouse anti-Flag antibodies (green). Nuclei were stained with DAPI. Scale bars = 10 µm. Data are presented as the mean ± SD of data from three independent experiments. *P < 0.05, **P < 0.01, ***P < 0.001, ****P < 0.0001.

To further delineate the interaction between ALIX and E2, cells were co-transfected with either the E2 mutant plasmid pFlag-E2-ΔYPXnL (lacking the YPXnL motif), pFlag-E2, or empty pFlag, along with pHA-ALIX. These cells were subjected to confocal microscopy and Co-IP assays. The findings revealed that ALIX specifically interacted with E2, while the E2 mutant lacking the YPXnL motif did not (Fig. 2A and B). Additionally, cells co-transfected with pFlag-E2 and pHA-ALIX were infected with CSFV (MOI = 0.5) for 36 h, after which whole cell lysates and supernatants were collected. RT-qPCR and virus titration assays demonstrated a significant decrease in viral mRNA levels and viral loads when the YPXnL motif was absent, even in the presence of HA-ALIX co-transfection. Furthermore, western blotting assay showed a significant downregulation of both intracellular and released E2 expressions in cells co-transfected with pFlag-E2-ΔYPXnL and pHA-ALIX, as well as in cells co-transfected with pFlag-vector and HA-ALIX. In contrast, cells co-transfected with the plasmids overexpressing full-length E2 and ALIX maintained normal levels of E2 expression in both intracellular compartments and supernatants, indicating an unaffected viral infection state (Fig. 2C). These results indicate that the YPXnL motif of E2 is essential for ALIX recognition. ALIX is composed of three domains: the N-terminal Bro1 domain (homologous to yeast Bro1), which is known to facilitate ESCRT-III binding; a central V domain, which has been reported to interact with the HIV Gag protein, thereby assuming a pivotal role in HIV budding; and a C-terminal proline-rich region (PRR, absent in crystal structures), which serves as a docking site for various interacting proteins, including those with TSG101 and SH3 domains (34). Each domain contributes distinct functional roles (Fig. 2D). To identify the critical domains involved in E2 recognition and binding, considering the porcine-derived cells used in the study, we selected the ALIX gene of *Sus scrofa* (A0A287AH24) as a reference. We constructed three plasmids expressing the individual ALIX domains: pHA-ALIX-Bro1, pHA-ALIX-V, and pHA-ALIX-PRR. Cells were co-transfected with pFlag-E2 and either pHA-ALIX-Bro1, pHA-ALIX-V, or pHA-ALIX-PRR using full-length pHA-ALIX and an empty pHA vector as positive and negative controls, respectively, followed by confocal microscopy and Co-IP assays. The results revealed that only full-length ALIX and ALIX-Bro1 demonstrated significant co-localization and interaction with E2 (Fig. 2E and F). Given the substantial deviation of these findings from previous studies, we compared the human ALIX gene with that from *S. scrofa* and examined existing research on the predicted structural features of the ALIX domains. Leveraging this information, we engineered a series of plasmids encoding distinct human ALIX domains: pHA-ALIX, pHA-ALIX-Bro1 (1–358aa), pHA-ALIX-V (356–702aa), and pHA-ALIX (703–869aa). Co-IP and confocal microscopy assays were subsequently conducted, yielding results that exceeded our expectations. Both the Bro1 and V domains exhibited interactions with CSFV E2, albeit with differing affinities. Notably, Bro1 bound to Flag-E2 much more strongly than the V domain (Fig. S2). Taken together, these findings substantiate that the ALIX Bro1 domain specifically binds to the CSFV E2, ultimately influencing the budding of subviral particles.

### ALIX interacts with CSFV E2 in the Golgi apparatus for vesicular transport

**Fig 3:**
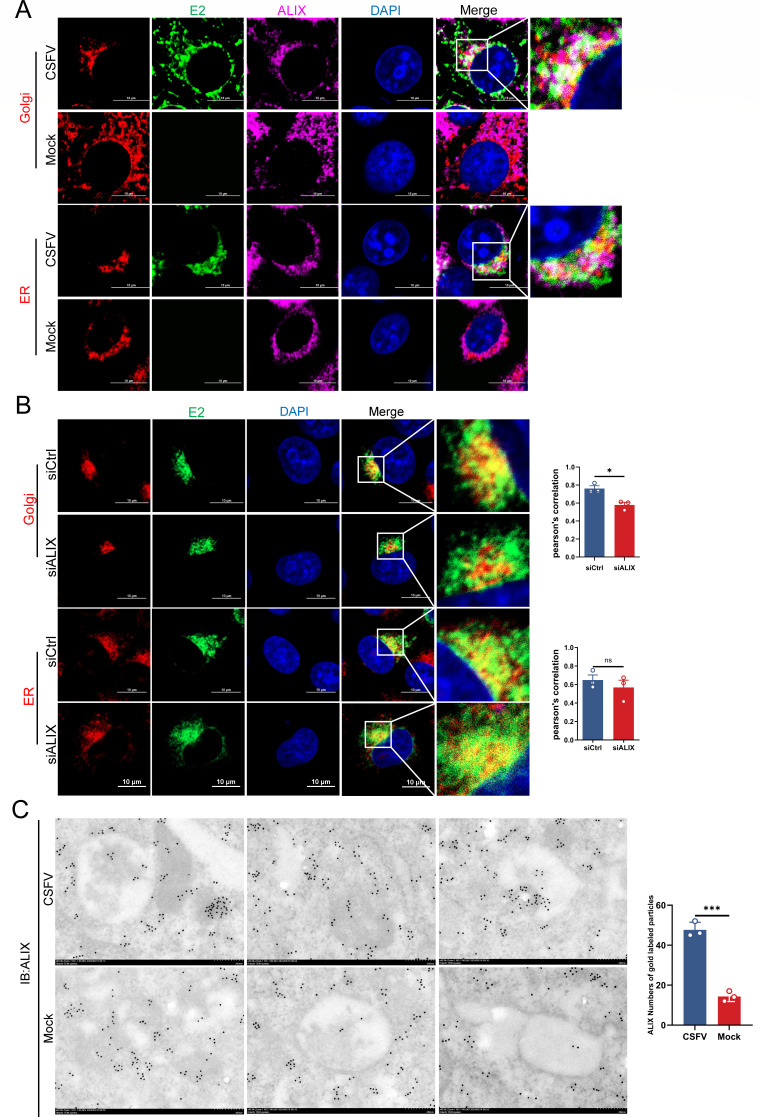
ALIX-E2 interaction in the Golgi apparatus mediates CSFV budding. (**A and B**) Cells infected with CSFV (MOI = 0.5) were incubated at 37°C, then fixed and stained with mouse anti-CSFV E2 monoclonal antibody (WH303, green), rabbit anti-ALIX antibody (purple), and Golgi marker (red) and observed by confocal microscopy. Nuclei were stained with DAPI. Scale bars = 10 µm. (**C**) Distribution of ALIX in infected cells by immunoelectron microscopy. Cells infected with CSFV (MOI = 0.5) for 36 h were fixed and stained with rabbit anti-ALIX antibody. Scale bar = 200 nm. These data are representative of three independent experiments. Data are presented as the mean ± SD of data from three independent experiments. *P < 0.05, **P < 0.01, ***P < 0.001.

To demonstrate the role of ALIX in interacting with E2 during the budding and transport stages from the ER membrane to the Golgi apparatus, infected cells were fixed and analyzed using confocal microscopy at 36 hpi. The fluorescent images revealed a more pronounced co-localization of endogenous ALIX with E2 in the Golgi compared to the ER (Fig. 3A). Following ALIX knockdown via targeted siRNA, confocal microscopy showed a significant reduction in the localization of E2 within the Golgi apparatus, with no notable changes in its positioning within the ER (Fig. 3B). Given that endosomal transport, a key pathway of the ESCRT system, plays a crucial role in the intracellular trafficking of viral particles (35), we focused on vesicle-like structures (36) to further explore the involvement of ALIX in CSFV particle budding. Immunoelectron microscopy results demonstrated that compared to the control group, ALIX gold particles significantly accumulated within intracellular vesicle-like structures postinfection. Quantitative analysis of multiple images from these regions confirmed a marked increase in ALIX aggregation in infected cells (Fig. 3C). Overall, these data highlight the role of ALIX and suggest that CSFV particles bud around the Golgi.

### ALIX recruits ESCRT-III for CSFV budding

**Fig 4:**
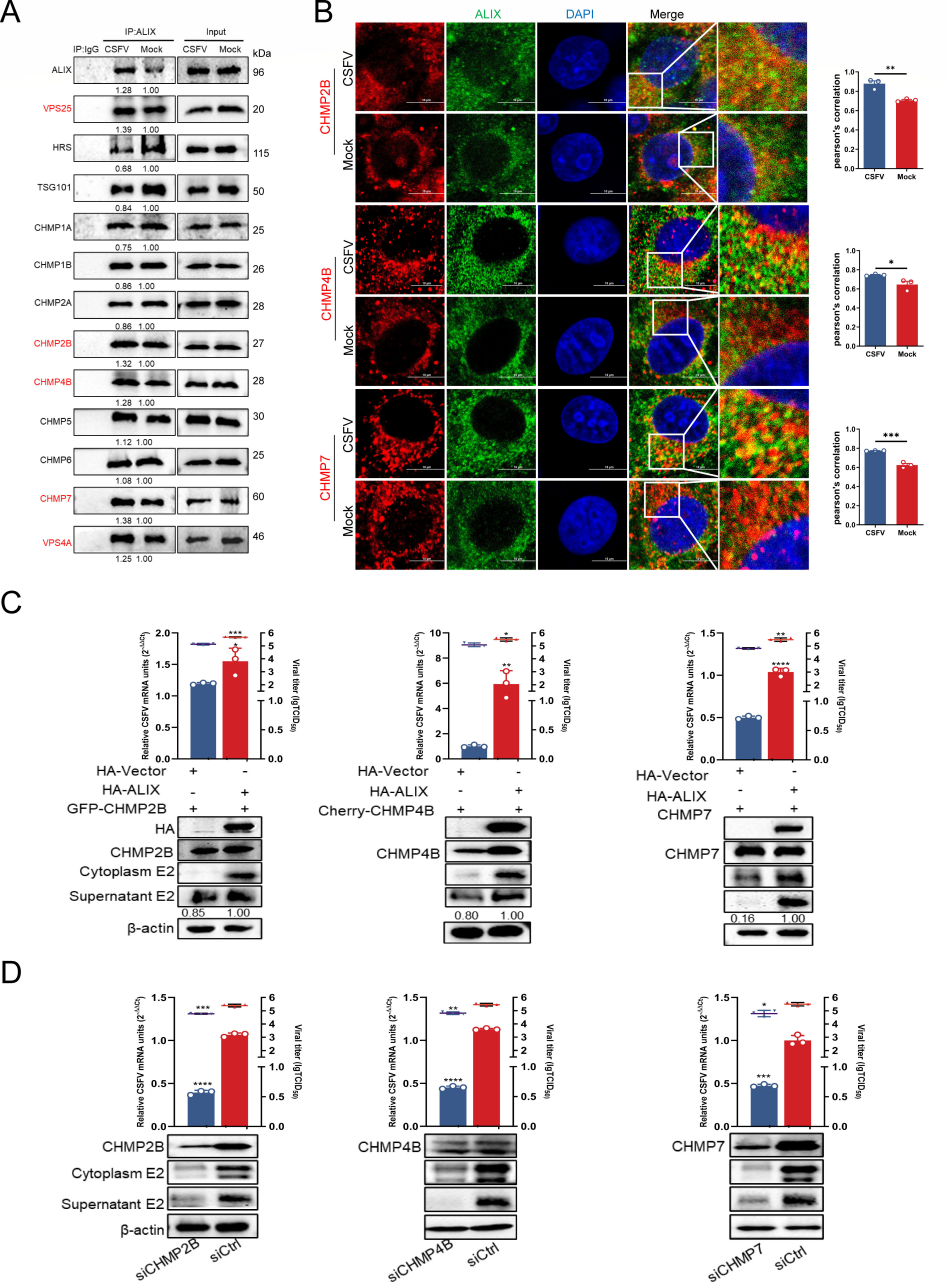
ESCRT-III assists ALIX in CSFV budding. (**A**) PK-15 cells infected with CSFV (MOI = 0.5) for 36 h were harvested and subjected to immunoprecipitation using rabbit anti-ALIX antibody. Whole-cell lysates were collected and subjected to western blotting using rabbit/mouse anti-ESCRT antibodies. These data are representative of three independent experiments. (**B**) CSFV-infected cells (MOI = 0.5) were fixed and stained using mouse anti-ALIX (green), rabbit anti-CHMP2B, rabbit anti-CHMP4B, and rabbit anti-CHMP7 antibodies (red). Nuclei were stained with DAPI. Scale bars = 10 µm. (**C**) PK-15 cells were transfected with indicated plasmids (pHA-ALIX, pGFP-CHMP2B, or pCherry-CHMP4B) or vector for 24 h, then infected with CSFV (MOI = 0.5) for 36 h. Harvested virus supernatant was used to reinfect fresh PK-15 cells for measuring viral titer. Cell cultures were harvested and subjected to RT-qPCR using the special primers and western blotting using rabbit anti-HA, rabbit anti-GFP, rabbit anti-Cherry, and rabbit anti-CHMP7 antibodies. (**D**) PK-15 cells were separately transfected with siCHMP2B, siCHMP4B, siCHMP7, or siCtrl for 24 h, then infected with CSFV (MOI = 0.5) for 36 h. The cell supernatant after re-infection was collected, and the virus titer was measured using titer titration (TCID_50_/mL). Cell cultures were harvested for RT-qPCR using the special primers and western blotting using rabbit anti-CHMP2B/CHMP4B/CHMP7 and mouse anti-E2 antibodies. These data are representative of three independent experiments. Data are presented as the mean ± SD of data from three independent experiments. *P < 0.05, **P < 0.01, ***P < 0.001.

**Fig 5:**
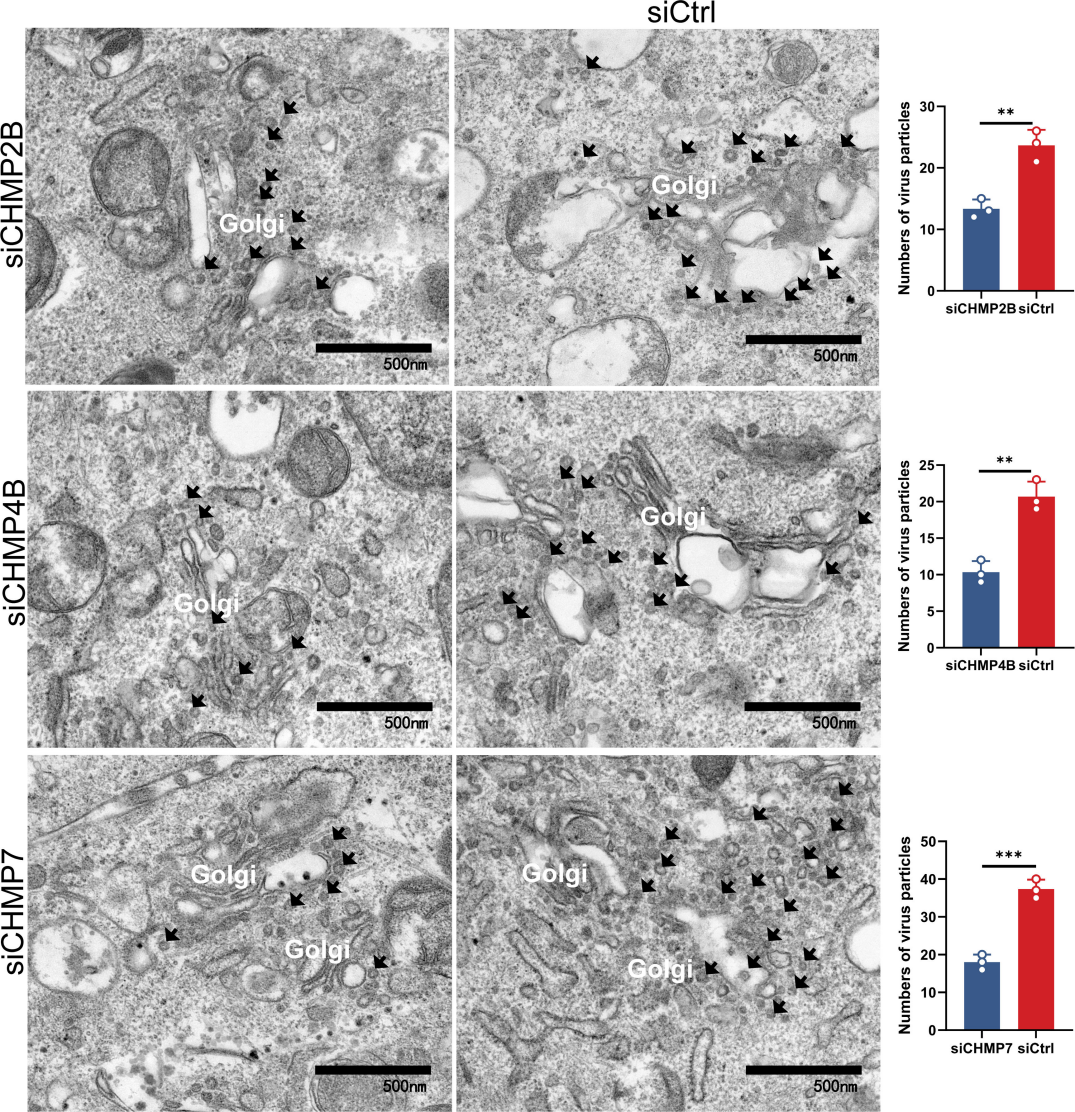
ESCRT-III participates in CSFV budding around the Golgi apparatus. Cells transfected with siCHMP2B, siCHMP4B, siCHMP7, or siCtrl were infected with CSFV (MOI = 0.5) for 36 h, and then subjected to TEM for the numbers of viral particles. These data are representative of three independent experiments. Data are presented as the mean ± SD of data from three independent experiments. **P < 0.01, ***P < 0.001.

ALIX not only recognizes viral particles but also facilitates vesiculation, a process critically dependent on the remodeling and membrane scission activities of ESCRT-III (37). To identify which ESCRT-III subunits are involved in ALIX-mediated vesiculation during CSFV budding, we compared the expression levels of ESCRT-III subunits interacting with ALIX between infected and mock cells. Co-IP assay revealed a significant increase in the interaction of ESCRT-III subunits CHMP2B, CHMP4B, and CHMP7 with ALIX upon infection compared to mock cells (Fig. 4A). Confocal microscopy further corroborated these findings, showing a markedly enhanced co-localization of ALIX with CHMP2B, CHMP4B, or CHMP7 in infected cells (Fig. 4B). To further investigate whether the enhanced interaction between ALIX and ESCRT-III subunits (CHMP2B, CHMP4B, CHMP7, VPS4A, and VPS25) was related to their increased association with ALIX, we conducted additional experiments at different infection time points. The recruitment of CHMP2B, CHMP4B, and CHMP7 to ESCRT-III by ALIX was notably stronger at 36 hpi, which was selected as the key time point for the study (Fig. S3A). Infected cells were treated with the intracellular vesicle synthesis inhibitor GW4869 (10 µM). Western blotting analysis revealed a significant reduction in the expression levels of CHMP2B, CHMP4B, CHMP7, VPS25, and VPS4A, as quantified by grayscale analysis (Fig. S3B). These findings indicate that the intracellular vesicle trafficking pathway plays a pivotal role in CSFV budding, and that ALIX along with the ESCRT-III subunits CHMP2B, CHMP4B, and CHMP7 are involved in vesicle formation and maturation. To further validate these interactions, cells were co-transfected with pHA-ALIX or pHA vector, along with pGFP-CHMP2B, pGFP-CHMP4B, or pGFP-CHMP7, and subsequently infected with CSFV (MOI = 0.5). At 24 hpi, supernatants were collected for reinfection experiments, and whole-cell lysates were harvested for further analysis. Western blotting results demonstrated a significant upregulation in E2 protein expression in cells transfected with pHA-ALIX alone or co-transfected with pHA-ALIX and pGFP-CHMP2B, -CHMP4B, or -CHMP7 compared to cells co-transfected with pHA vector and pGFP-CHMP2B, pCHMP4B, or CHMP7. The supernatants from these infected cells also exhibited a significant increase in E2 protein expression upon reinfection. Moreover, RT-qPCR and virus titration data from the supernatants indicated that transfection with ALIX alone, or in combination with CHMP2B, CHMP4B, or CHMP7, led to significant increases in CSFV mRNA levels and virus loads (Fig. 4C). Intriguingly, CHMP2B, CHMP4B, and CHMP7, along with CSFV NS4B, NS3, and NS5A, are implicated in the formation of VRC upon infection (23), implying that CSFV replication necessitates ESCRT-III. To further validate the role of ESCRT-III in CSFV budding, infected cells transfected with pFlag-E2 were fixed and stained with specific antibodies for confocal microscopy analysis. Results revealed a significant enhancement in the co-localization of endogenous CHMP2B and CHMP4B with exogenous E2 in infected cells compared to mock cells. Conversely, no significant change in the co-localization of CHMP2A with E2 was observed, irrespective of the infection status (Fig. S4). These findings strongly suggested that ALIX recruits ESCRT-III to facilitate CSFV budding. Additionally, cells treated with siRNA targeting CHMP2B, CHMP4B, or CHMP7 (siCHMP2B, siCHMP4B, or siCHMP7) or siCtrl for 24 h were subsequently infected with CSFV (MOI = 0.5). Supernatants were collected at 36 hpi for reinfection studies. Western blotting assay of both whole-cell lysates and supernatants revealed significantly reduced levels of E2 expression in cells treated with siCHMP2B, siCHMP4B, or siCHMP7 compared to siCtrl-treated cells. RT-qPCR and virus titration assays further demonstrated significant reduction in CSFV mRNA levels and progeny virus loads following the knockdown of these ESCRT-III subunits (Fig. 4D). Remarkably, TEM result showed a substantial decrease in virus particles clustered near the Golgi apparatus in cells treated with siCHMP2B, siCHMP4B, or siCHMP7 compared to those treated with siCtrl (Fig. 5). These findings suggest that ESCRT-III subunits recruited by ALIX significantly enhance the efficiency of virus budding. Furthermore, confocal microscopy revealed a marked increase in the fluorescence intensity of CHMP2B, CHMP4B, and CHMP7 localized in the Golgi apparatus of infected cells compared to mock-treated cells, whereas CHMP2A, serving as a negative control, showed no such increase (Fig. S5). Collectively, these data indicate that ALIX facilitates the vesicular transport of CSFV E2 to the Golgi apparatus and recruits CHMP2B, CHMP4B, and CHMP7 to support the budding of mature virus particles.

### Rab8 mediates ALIX-assisted CSFV vesicular transport

**Fig 6:**
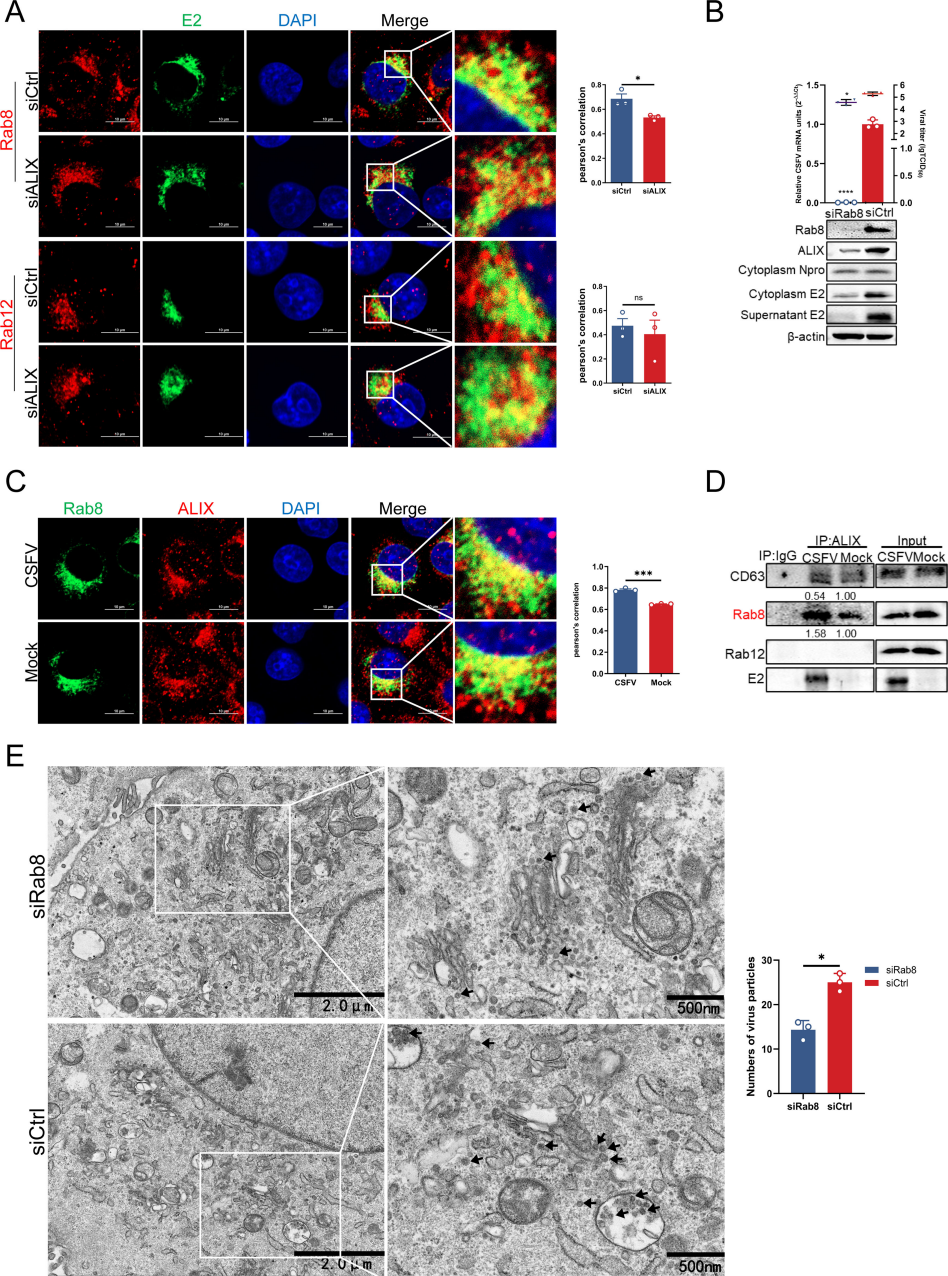
Rab8 mediates the transport of CSFV particles. (**A**) Cells treated with siALIX or siCtrl were infected with CSFV (MOI = 0.5) for 24 h, then fixed and subjected to confocal microscopy using mouse anti-E2 (green), rabbit anti-Rab8, or -Rab12 antibodies (red). Nuclei were stained with DAPI. Scale bars = 10 µm. (**B**) Cells treated with siRab8 or siCtrl were infected with CSFV (MOI = 0.5) for 36 h. The cell supernatants after reinfection were collected, and the virus titer was measured using titer titration (TCID_50_/mL). Cell cultures were harvested for RT-qPCR using the special primers and western blotting using rabbit anti-Rab8, rabbit anti-ALIX, rabbit anti-Npro, and mouse anti-E2 antibodies. (**C**) PK-15 cells infected with CSFV (MOI = 0.5) for 36 h were fixed and subjected to confocal microscopy using rabbit anti-Rab8 (green) and mouse anti-ALIX antibodies (red). Nuclei were stained with DAPI. Scale bars = 10 µm. (**D**) PK-15 cells were infected with CSFV (MOI = 0.5) for 36 h, and then harvested for immunoprecipitation using rabbit anti-ALIX antibody. Whole-cell lysates were harvested and subjected to western blotting using rabbit anti-CD63, rabbit anti-Rab8, rabbit anti-Rab12, and mouse anti-E2 antibodies. These data are representative of three independent experiments. (**E**) PK-15 cells were transfected with siRab8 or siCtrl for 24 h, and then infected with CSFV (MOI = 0.5) for 36 h. Viral particles around the Golgi apparatus were counted by transmission electron microscopy. Data are presented as the mean ± SD of data from three independent experiments. *P < 0.05, **P < 0.01, ***P < 0.001, ****P < 0.0001.

Subviral particles undergo assembly into mature virions within Golgi apparatus and are subsequently transported from the cis-Golgi to the trans-Golgi network for further processing (38, 39). We demonstrated that ALIX recognized and binded to E2 within the Golgi apparatus, thereby facilitating budding and transport with the assistance of ESCRT-III. However, the precise mechanism by which the ESCRT machinery mediates the transport of viral particles from the Golgi apparatus remains poorly understood. The vesicle transport pathway mediated by endosomes constitutes an efficient mechanism for viral particles to traverse the densely packed cytoplasm and localize to specific intracellular compartments essential for successful infection (40). Rab proteins are integral to vesicle transport and the regulation of endosomes maturation (41). To investigate this, we screened four Rab GTPases associated with the Golgi apparatus following ALX knockdown. The results indicated that co-localization of Rab8 with CSFV E2 was significantly diminished in siALIX-treated infected cells compared to siCtrl-treated infected cells. In contrast, there were no significant alterations in the co-localization of Rab12, Rab9, and Rab11 with E2 upon infection (Fig. 6A; Fig. S6A). Notably, Rab8 localization to the Golgi apparatus was significantly enhanced following infection (Fig. S6B). Additionally, we evaluated the expression levels of Rab8, Rab9, Rab11, and Rab12 in cell lysates collected at 12, 24, and 36 hpi using western blotting. The expression levels of these Rabs remained relatively unchanged throughout the course of CSFV infection, suggesting that Rab8-mediated vesicular transport aids in viral trafficking by modulating its localization rather than upregulating its expression (Fig. S7A). To further confirm the critical role of Rab8 in this transport process, cells transfected with siRab8 or siCtrl were infected with CSFV (MOI = 0.5). At 36 hpi, supernatants were collected and subjected to infect fresh cells. Western blotting result revealed that Rab8 knockdown significantly reduced the expression levels of ALIX and both intracellular and extracellular E2, alongside a marked reduction in CSFV mRNA levels and progeny virus titers (Fig. 6B). Moreover, a significant increase in the co-localization of Rab8 and ALIX in infected cells was detected compared to that in mock-treated cells (Fig. 6C). This interaction between Rab8 and ALIX was further substantiated by a Co-IP assay (Fig. 6D). TEM analysis following Rab8 knockdown demonstrated a notable decrease in virus particles near the Golgi apparatus and trans-Golgi vesicles, with a corresponding enrichment of viral particles around ER vesicles. This observation suggests that Rab8 facilitates viral transport around the Golgi membrane (Fig. 6E). In summary, these findings underscore the critical role of Rab8-regulated vesicle transport in the efficient budding of CSFV.

### Kinesin family member 4a (Kif4A) is responsible for vesicular transport

**Fig 7:**
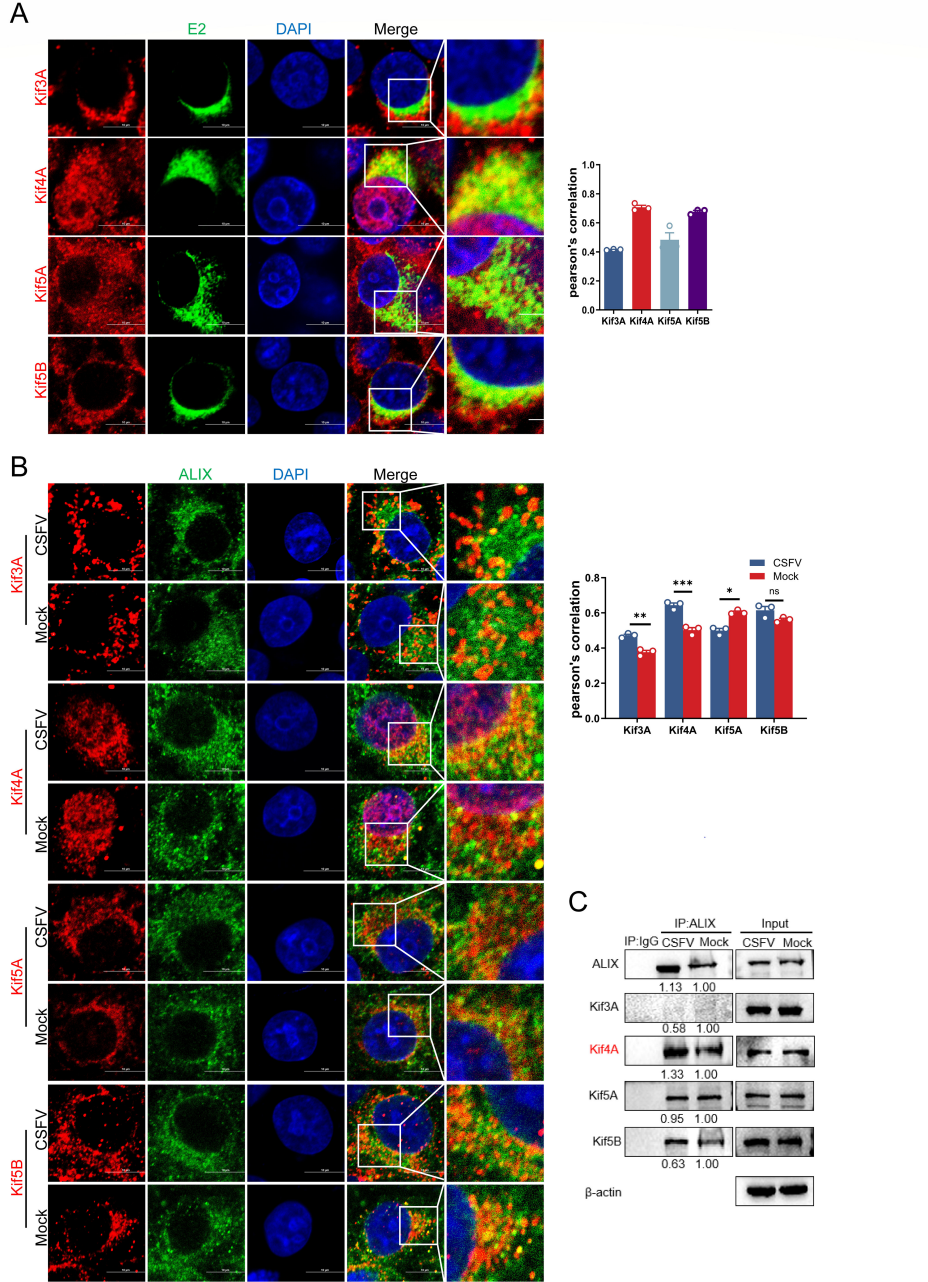
Kif4A is involved in CSFV budding. (**A**) PK-15 cells infected with CSFV (MOI = 0.5) for 36 h were fixed and subjected to confocal microscopy using rabbit anti-Kif3A/Kif4A/Kif5A/Kif5B (red) and mouse anti-E2 antibodies (green). Nuclei were stained with DAPI. Scale bars = 10 µm. (**B**) Cells infected with CSFV (MOI = 0.5) for 36 h were fixed and stained with mouse anti-ALIX (green) and rabbit anti-Kif3A/Kif4A/Kif5A/Kif5B antibodies (red). Nuclei were stained with DAPI. Scale bars = 10 µm. (**C**) PK-15 cells infected with CSFV (MOI = 0.5) for 36 h were harvested and subjected to immunoprecipitation using rabbit anti-ALIX antibody. Whole-cell lysates were harvested and subjected to western blotting using rabbit anti-Kif3A/Kif4A/Kif5A/Kif5B antibodies, as well as rabbit anti-tubulin and mouse anti-vimentin antibodies. Data are presented as the mean ± SD of data from three independent experiments. *P < 0.05, **P < 0.01, ***P < 0.001.

**Fig 8:**
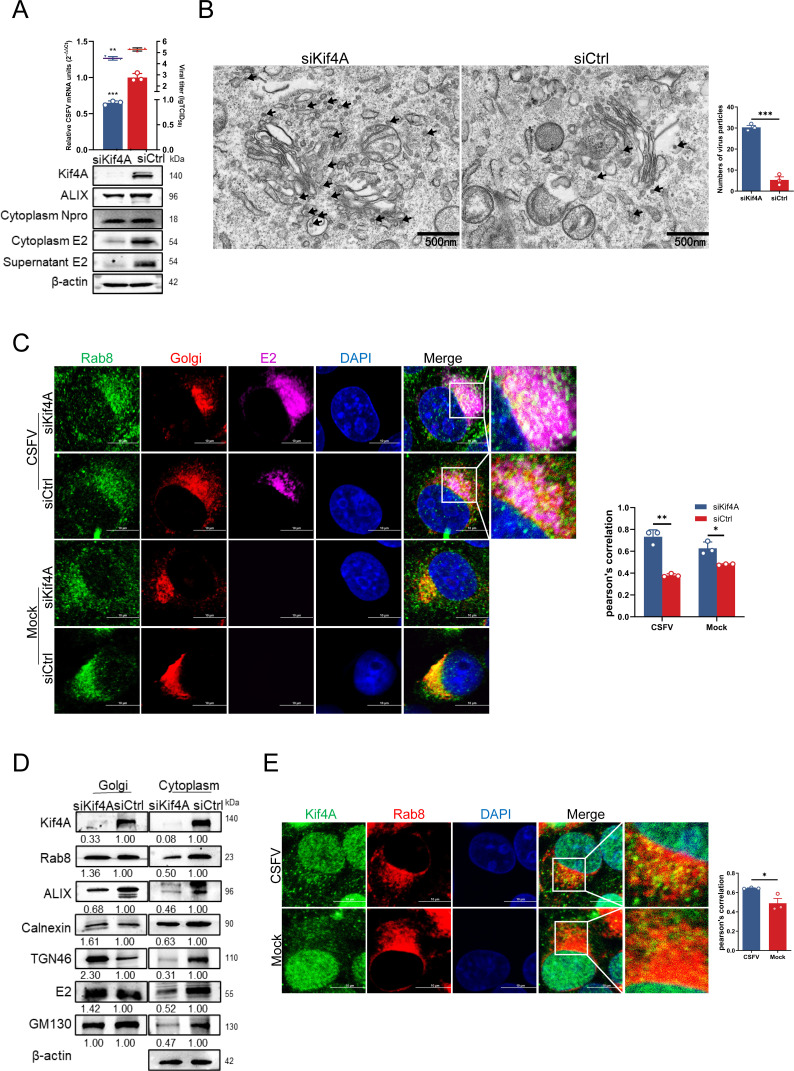
Kif4A transports the positive vesicles mediated by Rab8. (**A**) Cells transfected with siKif4A or siCtrl were infected with CSFV (MOI = 0.5) for 36 h. The cell supernatants after reinfection were collected, and the virus titer was measured using titer titration (TCID_50_/mL). Cell cultures were harvested for RT-qPCR using the special primers and western blotting using rabbit anti-Rab8, rabbit anti-ALIX, rabbit anti-Npro, and mouse anti-E2 antibodies (**B**). PK-15 cells transfected with siKif4A or siCtrl for 24 h were infected with CSFV (MOI = 0.5) for 36 h. Viral particles around the Golgi apparatus were counted by transmission electron microscopy. These data are representative of three independent experiments. (**C**) PK-15 cells transfected with siKif4A or siCtrl were infected with CSFV (MOI = 0.5) for 36 h, then fixed and stained with rabbit anti-Rab8 (green) and mouse anti-E2 antibodies (purple), and Golgi-Tracker Red for confocal microscopy. Nuclei were stained with DAPI. Scale bars = 10 µm. (**D**) Cells infected with CSFV (MOI = 0.5) for 36 h were harvested and subjected to Golgi apparatus extraction. Whole-cell lysates were harvested and subjected to western blotting using rabbit anti-Kif3A, anti-Kif4A, anti-Kif5A, anti-Kif5B, rabbit anti-Rab8, anti-ALIX, anti-Calnexin, anti-TGN46, anti-Npro, and anti-GM130 antibodies. (**E**) PK-15 cells infected with CSFV (MOI = 0.5) for 36 h were fixed and stained with mouse anti-Kif4A (green) and rabbit anti-Rab8 (red) for confocal microscopy. Nuclei were stained with DAPI. Scale bars = 10 µm. Data are presented as the mean ± SD of data from three independent experiments. *P < 0.05, **P < 0.01, ***P < 0.001.

Specific kinesin family proteins are essential for intracellular transport from the negative to the positive pole (42, 43). Kinesins leverage microtubule polarity to facilitate the movement of cargo toward the positive pole, thereby directing it from the cell center outward (44). To identify the kinesin proteins involved in transporting ALIX-coated vesicles containing CSFV particles, we screened Kif3A (Kinesin-2 family), Kif4A (Kinesin-4 family), Kif5A, and Kif5B (Kinesin-1 family) (45–47). First, western blotting assay of cell lysates collected at 12, 24, and 36 hpi revealed a significant upregulation of Kif4A and Kif5B expression levels at 24 hpi (Fig. S7B). Confocal microscopy of infected cells stained with specific antibodies demonstrated that Kif4A and Kif5B co-localized with CSFV E2 (Fig. 7A). Furthermore, confocal microscopy and Co-IP assays were conducted to assess the interactions between the four kinesin proteins and ALIX during infection. The results indicated a marked enhancement in the interaction between Kif4A and ALIX under CSFV infection (Fig. 7B and C), implicating the important role of Kif4A in the CSFV infection process. Previous studies have shown that Kif5B is involved in the transport of multiple vesicles post-endocytosis (48). We hypothesized that Kif4A may play a novel role in virion budding. To validate the role of Kif4A in CSFV budding, Kif4A knockdown cells were infected with CSFV (MOI = 0.5) for 36 h. Supernatants were then collected and used to infect fresh cells. After 36 hpi, whole-cell lysates and supernatants were harvested for subsequent analysis. Western blotting result demonstrated that both intracellular and extracellular E2 protein levels were markedly diminished following Kif4A knockdown, while intracellular Npro protein expression levels remained unaffected (Fig. 8A). TEM of siKif4A-treated infected cells revealed a pronounced increase in the aggregation of CSFV particles around the Golgi apparatus (Fig. 8B). Confocal microscopy further investigated that Kif4A knockdown significantly enhanced the co-localization of Rab8 with CSFV E2 within the Golgi apparatus (Fig. 8C). Additionally, Golgi fractions from siKif4A-treated infected cells exhibited elevated Rab8 expression within the Golgi apparatus and reduced levels in the cytoplasm. The upregulation of the trans-Golgi network protein TGN46 suggested that Kif4A knockdown impedes retrograde Golgi vesicle trafficking toward the plasma membrane, resulting in the accumulation of Rab8/ALIX vesicles within the Golgi apparatus (Fig. 8D). Upon comparing the confocal microscopy images of infected and Mock groups, it was observed that the co-localization of Kif4A and Rab8 in the cytoplasm was markedly increased following CSFV infection (Fig. 8E). In conclusion, the compiled data suggest that during CSFV budding, Rab8-mediated vesicle transport from the Golgi apparatus to the plasma membrane is predominantly facilitated by Kif4A.

Besides, ESCRT-III key subunits are integral to the vesicular transport of viral particles. To elucidate the interaction between ESCRT-III and Kif4A, infected cells were fixed and stained with specific antibodies for confocal microscopy. The images demonstrated a distinct co-localization between Kif4A and CHMP4B, whereas no such co-localization was observed with Kif3A, Kif5A, or Kif5B (Fig. S8A). This interaction was further substantiated by Co-IP assay (Fig. S8B). This suggests that the markedly enhanced interaction between CHMP4B and Kif4A during CSFV infection further substantiates involvement of Kif4A in the transport of ALIX-associated vesicles.

### Microtubules participate in Rab8-mediated vesicle transport

**Fig 9:**
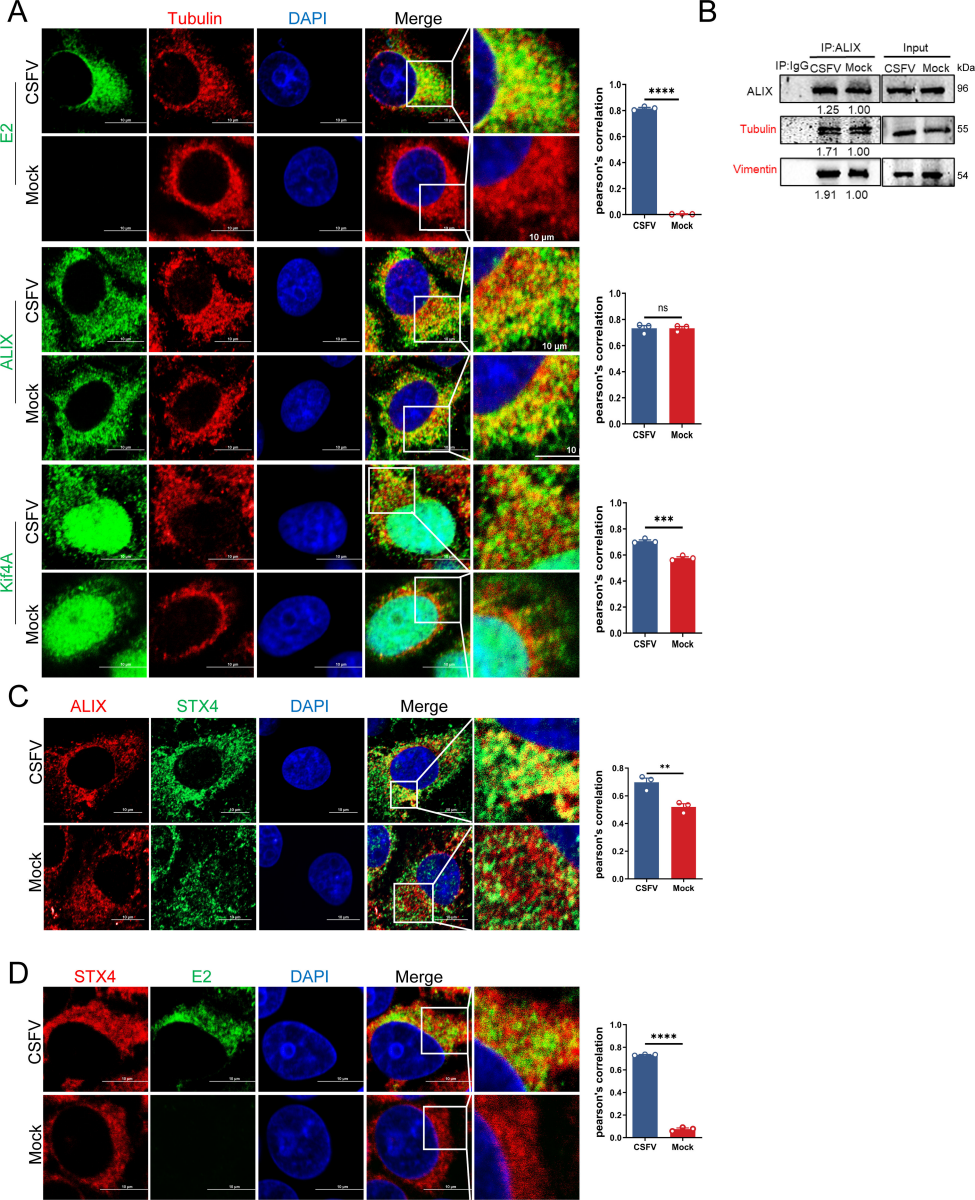
Microtubules participate in CSFV budding. (**A**) Cells infected with CSFV (MOI = 0.5) for 36 h were fixed and stained with rabbit anti-tubulin (red), mouse anti-ALIX (green), mouse anti-E2 (green), and mouse anti-Kif4A antibodies (green) for confocal microscopy. Nuclei were stained with DAPI. Scale bars = 10 µm. (**B and C**) PK-15 cells infected with CSFV (MOI = 0.5) for 36 h were fixed and subjected to confocal microscopy using mouse anti-ALIX (red), rabbit anti-STX4 (green) (**B**), rabbit anti-STX4 (red), and mouse anti-E2 antibodies (green) (**C**). Nuclei were stained with DAPI. Scale bars = 10 µm. Data are presented as the mean ± SD of data from three independent experiments. *P < 0.05, **P < 0.01, ***P < 0.001, ****P < 0.0001.

Motor proteins facilitate the loading and transported cargoes, such as inclusions and vesicles, along microtubules toward the plasma membrane, enabling viral budding (49). Confocal microscopy demonstrated substantial co-localization of tubulin with CSFV E2 at 36 hpi, along with increased co-localization of tubulin with ALIX or Kif4A upon infection (Fig. 9A). To further investigate the role of microtubules, infected cells were fixed and stained with the specified antibodies. Co-IP assay revealed that the interactions between microtubules and ALIX were significantly enhanced following CSFV infection, suggesting that microtubules may play a critical role in the transport of ALIX-associated vesicles carrying CSFV particles (Fig. 9B). To further investigate the role of microtubules, infected cells were fixed and stained with the specified antibodies. Moreover, Syntaxin 4 (STX4), a SNARE family membrane protein primarily localized at the plasma membrane (50, 51), regulates vesicle fusion by forming complexes with other SNARE proteins, such as SNAP-23, thereby facilitating the fusion of vesicles with the plasma membrane to expel intracellular substances into the extracellular space (52). To this end, infected cells were fixed and stained with specific antibodies for confocal microscopy. The images confirmed that ALIX exhibited significantly enhanced co-localization with STX4, and STX4 also co-localized with CSFV E2 (Fig. 9C and D). These findings suggest that mature CSFV particles may exploit the ALIX-mediated vesicle transport pathway to fuse with the plasma membrane for budding. Collectively, these results imply that microtubules function as conduits for virus budding.

## DISCUSSION

**Fig 10:**
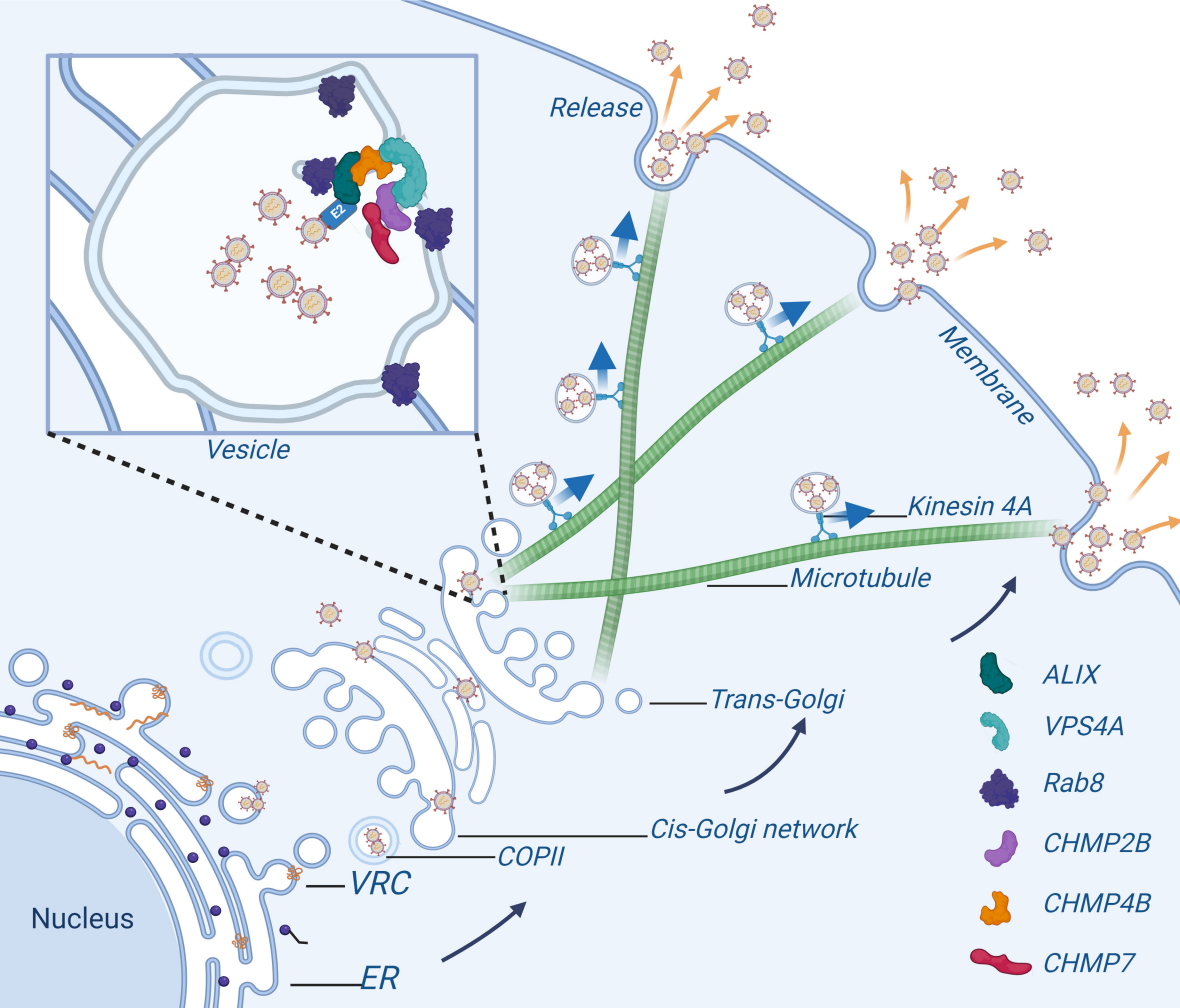
Schematic model depicting the role of ALIX and ESCRT-III in CSFV budding. ALIX interacted with CSFV E2 and recruited ESCRT-III subunits CHMP2B, CHMP4B, and CHMP7, facilitating the transport of CSFV-positive vesicles (2). Rab8 regulated the viral budding of mature vesicles on the Golgi apparatus (3). Kif4A loaded Rab8/ALIX vesicles to assist in the vesicular budding and transport of CSFV virions (4). Viral budding was transported along microtubules from Golgi apparatus to the cell membrane for release.

Extensive studies have elucidated that enveloped viruses co-opt the host cellular ESCRT machinery to facilitate viral particle egress via budding (9, 53). The role of the ESCRT system in modulating viral budding was initially delineated in retroviral infections (54, 55). More recently, members of the *Flavivirus* genus, such as JEV and DENV, have been shown to necessitate the ESCRT system for their egress from host cells (56). However, the intricate molecular mechanisms through which the ESCRT complex orchestrates membrane envelopment, vesicular transport, and the budding of flaviviruses remain poorly characterized. In this study, we investigated the intracellular dynamics of the critical ESCRT component ALIX and identified its interaction with the envelope glycoprotein E2 of CSFV. Our findings pinpoint the YPXnL late domain within E2 as a pivotal motif for ALIX recognition, essential for its binding to viral particles and mediating their vesicular trafficking. ALIX, in conjunction with Rab8, directs the transport of virus particles through the trans-Golgi network. Furthermore, with the involvement of Kif4A, these vesicular bodies are conveyed along microtubules to the plasma facilitating viral budding (Fig. 10). This study elucidates the ESCRT-dependent vesicular budding mechanism of CSFV, providing robust evidence that ESCRT subunits are critical mediators in the vesicular budding and trafficking processes, ultimately enabling the release of viral progeny.

In our preliminary investigations, we identified that ESCRT subunits engaged with the non-structural proteins of CSFV, significantly modulating its replication and synthesis within the ER. Post-replication, unassembled viral particles are shuttled in vesicles from the ER to the Golgi apparatus for further processing (57, 58). Viral particles undergo maturation within the Golgi apparatus and are subsequently trafficked along the trans-Golgi network (59). Throughout this process, the ESCRT-III complex orchestrates the protein-protein interactions necessary for the scission and detachment of vesicles from the donor membrane. Numerous enveloped viruses, including retroviruses, exploit the ESCRT pathway to mediate their egress via budding, which is essential for the abscission of intercellular bridges and the formation of microvesicles (60–62). For instance, the Equine Infectious Anemia Virus (EIAV) leverages ESCRT components such as ALIX, CHMP4B, CHMP2A, and either VPS4A or VPS4B during its budding process. In our study, we delineated the pivotal role of ALIX in recognizing the CSFV E2 protein, thereby facilitating vesicular transport during the CSFV budding process.

In this research, the ALIX Bro1 domain has been characterized as the binding interface for the CSFV YPXnL motif. Prior study has unveiled that the V domain of ALIX directly interacts with the YPXnL motif in HIV. It remains possible that the ALIX V domain engages with the YPXnL motif in CSFV, given the exceedingly low expression level of HA-ALIX-V observed in our Co-IP assay. Additionally, overexpression of the Bro1 domain may perturb endogenous ALIX complexes, which are known to form multimers within cells (63). Furthermore, our analysis of various CSFV and BDV isolate strains revealed the presence of the YPXnL motif, suggesting that this late domain is conserved across the *Pestivirus* genus. This hints that ALIX may have a conserved and essential function in the budding process of *Pestiviruses*.

ESCRT-III subunits CHMP2B, CHMP4B, and CHMP7 were integral participants in this process. Distinct from most retroviruses, CHMP7 played a crucial role in assisting ALIX during the vesicular transport of viral particles during CSFV budding. In contrast, CHMP5, which is implicated in HIV budding (64), exhibited no significant impact on its role in ALIX-mediated vesicular transport of CSFV particles (65). Rab proteins are key regulators of intracellular membrane and vesicle trafficking (66), orchestrating processes, such as vesicle biogenesis, directional transport (67), and fusion, to ensure precise intracellular cargo delivery (68–70). Unlike the well-characterized Rab5 and Rab7, Rab8 was investigated to significantly influence the morphology of the Golgi apparatus and critically involved in vesicle transport (71, 72). In this study, following CSFV synthesis and assembly within the VRC of the ER, viral particles were shed from the ER. ALIX recognized E2 YPXnL motif within the cytoplasm and, in conjunction with ALIX/ESCRT-III, facilitated the transport of vesicles to the Golgi apparatus. CSFV particles underwent further maturation within the Golgi, where vesicle differentiation occurred at the trans-Golgi network. Rab8-GTP localized at the trans-Golgi interacted with the motor protein effector Kif4A, enabling the anchoring of ALIX-containing vesicles laden with CSFV particles to the target membrane, namely, the plasma membrane. This coordination interaction promoted the microtubule-mediated transport of vesicles toward the plasma membrane.

Furthermore, West Nile virus (WNV) particles are assembled and egress from infected cells via the secretory pathway. RNAi analysis revealed that Rab8b plays a pivotal role in WNV particle budding by facilitating transport from the endocytic recycling compartment to the plasma membrane (69). During dengue virus and hantavirus infections, Rab8 colocalizes with the endocytic recycling protein Rab11 and viral proteins (73). Remarkably, reduced budding of infectious viral particles is observed only upon single or combined knockdown of Rab11a and Rab11b (69, 74). In this study, infected cells were examined for colocalization of Rab protein with E2 using confocal microscopy, which revealed an indispensable reduction in Rab8 and E2 colocalization under ALIX knockdown conditions. Targeted knockdown of Rab8 led to a markedly decreased intracellular and extracellular virus particle detection. The Rab8/ALIX-regulated vesicular transport system was identified to assist in the delivery of CSFV particles to the trans-Golgi network and subsequently to the plasma membrane for viral budding. Additionally, Kif4A knockdown furthered the accumulation of Rab8 and TGN46 at the trans-Golgi network, indicating a disruption in Rab8-mediated vesicle transport to the plasma membrane. This study, for the first time, establishes the involvement of the Kinesin-4 family, specifically Kif4A, in the intracellular transport processes associated with viral budding. Previous research has extensively documented the role of the Kinesin-1 family proteins (Kif5A, Kif5B, Kif5C, etc.) in viral replication and propagation (75, 76). These proteins are instrumental in facilitating the rapid intracellular transport of viral particles in host cells, including those of HSV (77), rabies (78), hepatitis (79), and influenza viruses (80), and CSFV (48, 81). Elucidating the role of Kif4A in CSFV budding provides novel insights and potential therapeutic avenues for combating CSFV.

This study unveils a novel role of the ESCRT system in the vesiculation and transport phases following CSFV replication, thereby deepening our understanding of the CSFV life cycle. Furthermore, the findings elucidate how membrane proteins of positive-sense RNA viruses can interact with host factors, such as the ESCRT complex, to facilitate specific budding and membrane remodeling, laying a critical foundation for exploring mechanisms to control viral budding.

## MATERIALS AND METHODS

### Virus and cell culture

The CSFV Shimen strain (GenBank accession number: AF092448) was utilized in this study, as described previously (22). Porcine kidney cells (PK-15) were cultured in Dulbecco’s Modified Eagle Medium (DMEM, GIBCO) supplemented with 10% fetal bovine serum (FBS) (GIBCO, Invitrogen), 0.2% NaHCO_3_, 100 µg/mL streptomycin, and 100 IU/mL penicillin (GIBCO, Invitrogen) at 37°C with 5% CO_2_.

### Plasmid construction

The pFLAG-E2, pFLAG-NS4B, and pFLAG-NS5A plasmids were generated by cloning corresponding CSFV genes into the p3 × FLAG-CMV-7.1 vector. The pHA-ALIX plasmid was constructed by cloning the ALIX gene into the pCAGGS-HA vector. Plasmids pCherry-ALIX(#21504), pGFP-CHMP2B (#115329), and pCherry-CHMP4B (#101850) were purchased from Addgene. The authenticity of each construct was confirmed by DNA sequencing.

### Transfection and RNA knockdown

PK-15 cells grown to 70% confluence on coverslip dishes were transfected with the indicated plasmid (2.5 µg) using Lipofectamine 3000 (Invitrogen) following the manufacturer’s protocol. At 6 hpt, the transfection medium was replaced with DMEM containing 2% FBS, and the cells were incubated for an additional 24 h. For RNA knockdown experiments, PK-15 cells were transfected with siRNA using Lipofectamine RNAiMAX in accordance with the manufacturer’s instructions. The siRNA duplexes and negative control siRNA were synthesized by Sangon. At 24 hpt, cells were infected with CSFV, and viral replication was assessed via RT-qPCR. Supernatant samples from progeny virus reinfection were collected, and viral particle sedimentation was analyzed by western blotting and confocal fluorescence microscopy at 36 hpi. The sequences of all corresponding siRNAs are listed in Table S2.

### Virus purification

PK-15 cells were transfected with plasmids or siRNAs for 24 h, followed by infection with CSFV for 36 h. Fresh cells were then re-infected with the collected supernatant samples for an additional 36 h. Offspring viruses were purified using PEG6000 and NaCl through the following steps: (i) adding NaCl to a final concentration of 0.5 mol/L and 10% PEG6000; (ii) incubating the mixture at 4°C overnight for at least 6 h; (iii) centrifuging at 13,000 × *g* for 30 min, collecting the precipitated virus, and resuspending it in an appropriate volume of PBS; (iv) centrifuging again at 13,000 × *g* for 1 h; and (v) the resulting precipitate constitutes the concentrated virus. Samples were prepared for western blotting by adding 1× SDS loading buffer.

### Confocal microscopy

PK-15 cells cultured on dishes were infected with CSFV (MOI = 0.5) and incubated at 37°C for 36 h. Following incubation, the cell monolayers were fixed with 4% paraformaldehyde (PFA) in PBS and permeabilized with 0.1% Triton X-100. For the visualization of CSFV and ESCRT subunits, cells were stained with mouse anti-CSFV E2 (WH303) and rabbit anti-ESCRT antibodies. To assess the colocalization of ESCRT with ER, Golgi, or Rab proteins, cells were stained with rabbit or mouse anti-ALIX antibody (Proteintech or Santa Cruz), rabbit anti-CHMP2B, rabbit anti-CHMP4B, and rabbit anti-CHMP7 antibodies, ER-Tracker Red or Golgi-Tracker Red, and various anti-Rab antibodies. For visualizing the colocalization of kinesins and ALIX or Rab8, cells were stained with rabbit anti-Kif3A, Kif4A, Kif5A, Kif5B, mouse anti-Kif4A, and corresponding anti-ALIX or Rab8 antibodies. Colocalization coefficients were calculated using Nikon A1 confocal microscope software and expressed as Pearson’s correlation coefficient. The details of all corresponding antibodies are provided in Table S3.

### Co-immunoprecipitation (Co-IP) and Western blotting

HEK-293T cells were co-transfected with pFLAG plasmids (pFLAG-E2, pFLAG-NS4B, pFLAG-NS5A, or vector), pCherry-ALIX, or pHA plasmids (pHA-ALIX, pHA-ALIX-Bro1, pHA-ALIX-V, pHA-ALIX-PRR). At 48 h post-transfection (pt), cells were lysed in NP-40 lysis buffer (50 mM Tris–HCl, 150 mM NaCl, 1% NP40, 1 mM EDTA, 1 mM PMSF, 1 mM NaF, 1 mM Na_3_VO_4_, pH = 7.4) for 30 min at 4°C. Lysates were clarified by centrifugation at 1,000 × *g* for 10 min at 4°C. An aliquot (20%) of the supernatant was set aside for later analysis, while the remaining lysate (80%) was incubated with control IgG and 20 µL of Protein A/G PLUS-Agarose slurry (Santa Cruz) for 4 h at 4°C with rotation. Agarose beads were removed by centrifugation at 1,000 × *g* for 5 min at 4°C. The lysates were then incubated with mouse anti-FLAG or rabbit anti-HA antibody for 4–6 h at 4°C. Subsequently, Protein A/G PLUS-Agarose slurry was added and incubated for an additional 2 h. The agarose beads were collected, washed with NP-40 lysis buffer, resuspended in 2× SDS loading buffer, and subjected to SDS-PAGE, followed by western blot analysis. The details of all corresponding antibodies are provided in Table S3.

### Extraction of the Golgi apparatus

The extraction of the Golgi apparatus was performed using a commercial reagent kit following the manufacturer’s instructions. Briefly, cells were transfected with or without siKIf4A for 24 h and subsequently infected with CSFV. The cells were then washed with pre-cooled PBS. A total of 25 × 10^6^ cells were collected by centrifugation at 600 × *g*, washed once with cooled PBS, and extracted using the Minute Golgi Apparatus Enrichment Kit (Invent, #GO-037, United States) according to the provided protocol.

### Electron and immunoelectron microscopy

Samples were processed for transmission electron microscopy (TEM) as previously described (82). Briefly, cells were fixed for 4 h at room temperature in 4% PFA and 0.25% glutaraldehyde in 0.1 M phosphate buffer (pH 7.4), followed by washing with double-distilled water and dehydration through a graded ethanol series (30, 50, 70, 80, and 90%). The cells were then embedded in fresh 100% LR White resin using BEEM capsules. Ultrathin sections (80 nm) were cut using a Leica UCT ultramicrotome and mounted onto 400-mesh high-transmission grids. Sections were immunolabeled with a rabbit anti-ALIX antibody and 10 nm colloidal gold-conjugated goat anti-rabbit IgG, followed by staining with uranyl acetate. The grids were examined with a transmission electron microscope at 80 kV (JEM-100SX TEM; NEC, Tokyo, Japan).

### Animal experiment

Ten 6-week-old specific-pathogen-free Large White pigs were randomly assigned to two groups of five and housed separately in distinct rooms at the Animal Experiment Center of Nanjing Agricultural University. Prior to the experiment, serological testing confirmed the pigs were free of key pathogens, including CSFV, PRRSV, PCV2, FMDV, and PRV, using enzyme-linked immunosorbent assay kits from IDEXX Laboratories, Inc. and JBT Agency, supplemented by PCR/RT-PCR assays for virus detection. The infected group was oro-nasally challenged with the CSFV Shimen strain (10^5^ TCID_50_), while the negative control (NC) group received PBS. Daily assessments included monitoring rectal temperature, clinical scoring (1 point for no fever, 2 for mild clinical signs with fever, 3 for severe clinical signs, 4 for death), and conducting pathological examinations according to established protocols. On 9 days post-challenge (dpc), pigs exhibiting typical symptoms, such as high fever, diarrhea, and skin hemorrhages, were euthanized. Tissues, including the lymph nodes, spleen, kidney, lung, tonsil, heart, liver, and intestine, were collected for RT-qPCR and IHC assays.

### Immunohistochemistry (IHC)

Paraffin-embedded tissue sections were mounted onto glass slides and dried overnight at 37°C. After deparaffinization, antigen retrieval was performed using 0.01 M citric acid buffer. To block endogenous peroxidase activity, tissues were treated with 3% H₂O₂ in methanol for 10 min. The sections were then blocked with 3% BSA for 30 min at room temperature. Subsequently, the sections were incubated overnight at 4°C with a 1:500 dilution of anti-ALIX antibody. The following day, an HRP-conjugated secondary antibody was applied, followed by a 30 min incubation at room temperature. Tissues were stained with DAB solution for 2 min and counterstained with hematoxylin for an additional 2 min. After washing, the slides were dehydrated with ethanol, cleared with xylene, and examined under a microscope.

### Statistical analysis

All data were presented as means ± standard deviations (SD). Student’s *t*-test was used to compare treated and untreated groups. Statistical significance is indicated by asterisks (*P < 0.05, **P < 0.01, ***P < 0.001, ****P < 0.0001) in the figures. All statistical analyses and calculations were performed using Prism 6 (GraphPad Software, Inc., La Jolla, CA).

## SUPPLEMENTAL MATERIAL

Supplemental figures mbio.02618-24-s0001.docx Figs. S1–S8.

Supplemental tables mbio.02618-24-s0002.docx Tables S1 to S3.

ASM does not own the copyrights to Supplemental Material that may be linked to, or accessed through, an article. The authors have granted ASM a non-exclusive, world-wide license to publish the Supplemental Material files. Please contact the corresponding author directly for reuse.
