# Supplementary material for: Classical swine fever virus recruits ALIX and ESCRT-III to facilitate viral budding
Source: mBio. 2025 Feb 25;16(4):e02618-24. doi: 10.1128/mbio.02618-24 (PMC11980558; doi:10.1128/mbio.02618-24)

**Supporting information**

**S1 Fig. *Pestiviruses* including CSFV and BDV have YPXL motifs.**

(A) Sequence comparison of the corresponding positions on the genome of different CSFV and BDV standard strains revealed the YPXnL late domain (900-930 aa) as the conservative region. (B) Cells transfected with siALIX were infected with CSFV (MOI=0.5) for 36 h and whole-cell lysates were subjected to Western blotting using rabbit anti-ALIX, rabbit anti-Npro and mouse anti-E2 antibodies, along with β-actin as a loading control. Data are presented as the mean ± SD of data from three independent experiments. **p < 0.05, **p < 0.01*.

**
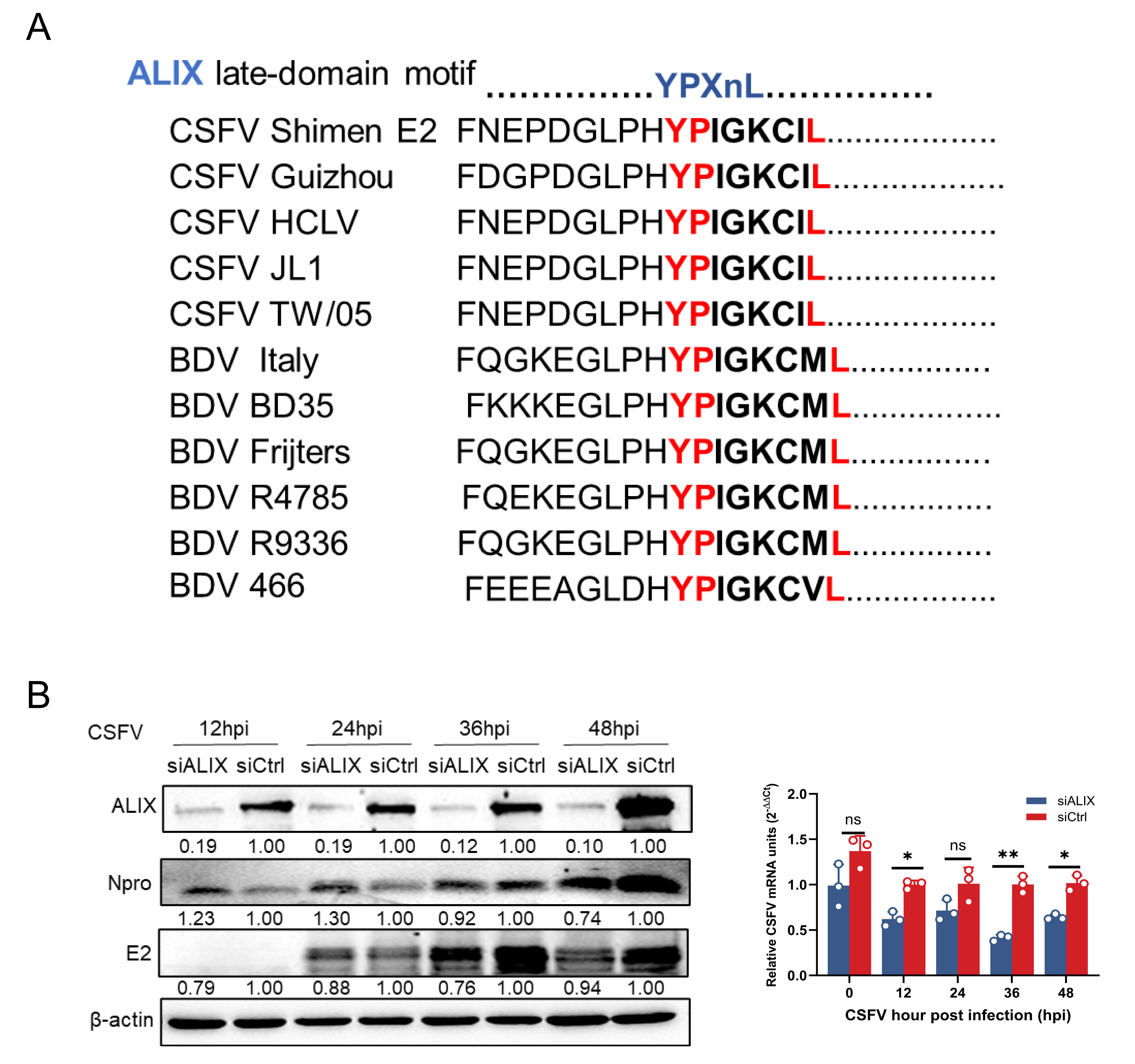
**

**S2 Fig. Human ALIX Bro1 and V domains interacts with the E2 of CSFV.**

(A) ALIX consists of three structural domains: the Bro1 domain (1-358aa), V domain (356-702aa), and PRR domain (703-869aa). (B) HEK-293T cells were co-transfected with the plasmids pFlag-E2 and pHA-ALIX, pHA-ALIX-Bro1, pHA-ALIX-V, or pHA-ALIX-PRR for 24 h, then harvested for immunoprecipitation using rabbit anti-HA antibody. Whole-cell lysates were harvested and subjected to Western blotting using rabbit anti-HA antibody or mouse anti-Flag antibody. (C) PK-15 cells co-transfected with the plasmids pFlag-E2 and pHA-ALIX, pHA-ALIX-Bro1, pHA-ALIX-V, or pHA-ALIX-PRR for 24 h were infected with CSFV (MOI=0.5) for 36 h. Cells were fixed and subjected to confocal microscopy using rabbit anti-ALIX antibody (red) and mouse anti-Flag antibody (green). Nuclei were stained with DAPI. Scale bars=10 μm. Data are presented as the mean ± SD of data from three independent experiments. ***p < 0.01, ***p < 0.001, ****p < 0.0001*.

**
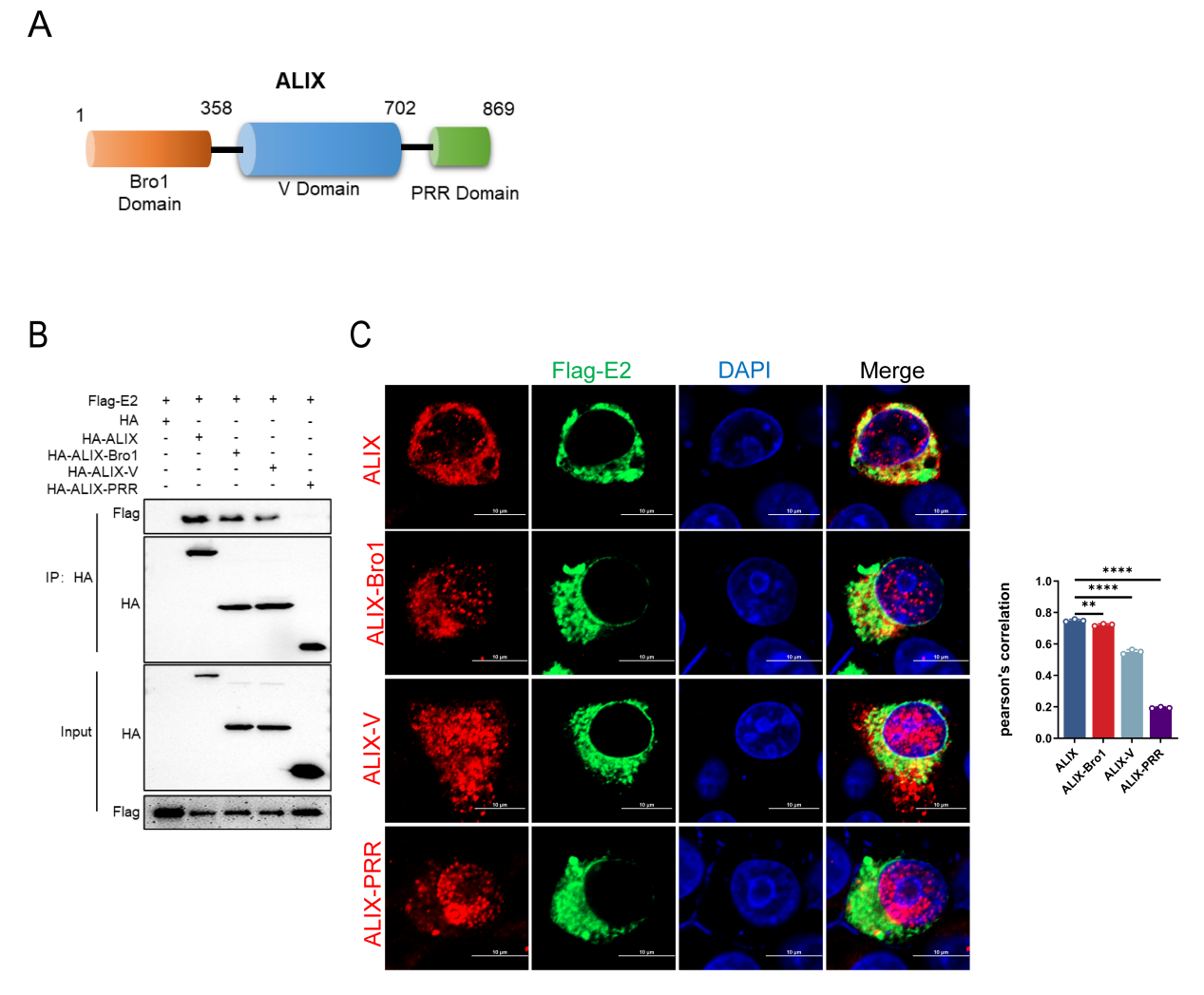
**

**S3 Fig. ESCRT-III assists ALIX in CSFV budding.**

(A) PK-15 cells infected with CSFV (MOI=0.5) for different time points were harvested and subjected to immunoprecipitation using rabbit anti-ALIX antibody. Whole-cell lysates were collected and subjected to Western blotting using rabbit anti-CHMP2B, anti-CHMP4B, anti-CHMP7, anti-VPS25, anti-VPS4A, anti-ALIX antibodies. (B) PK-15 cells were treated with GW4869 (10 µM) and subsequently infected with CSFV (MOI=0.5) for 36 h. Whole-cell lysates were collected and subjected to Western blotting using rabbit anti-Rab8, anti-ALIX, anti-CHMP4B, anti-CHMP7, anti-VPS25, anti-VPS4A antibodies.

**
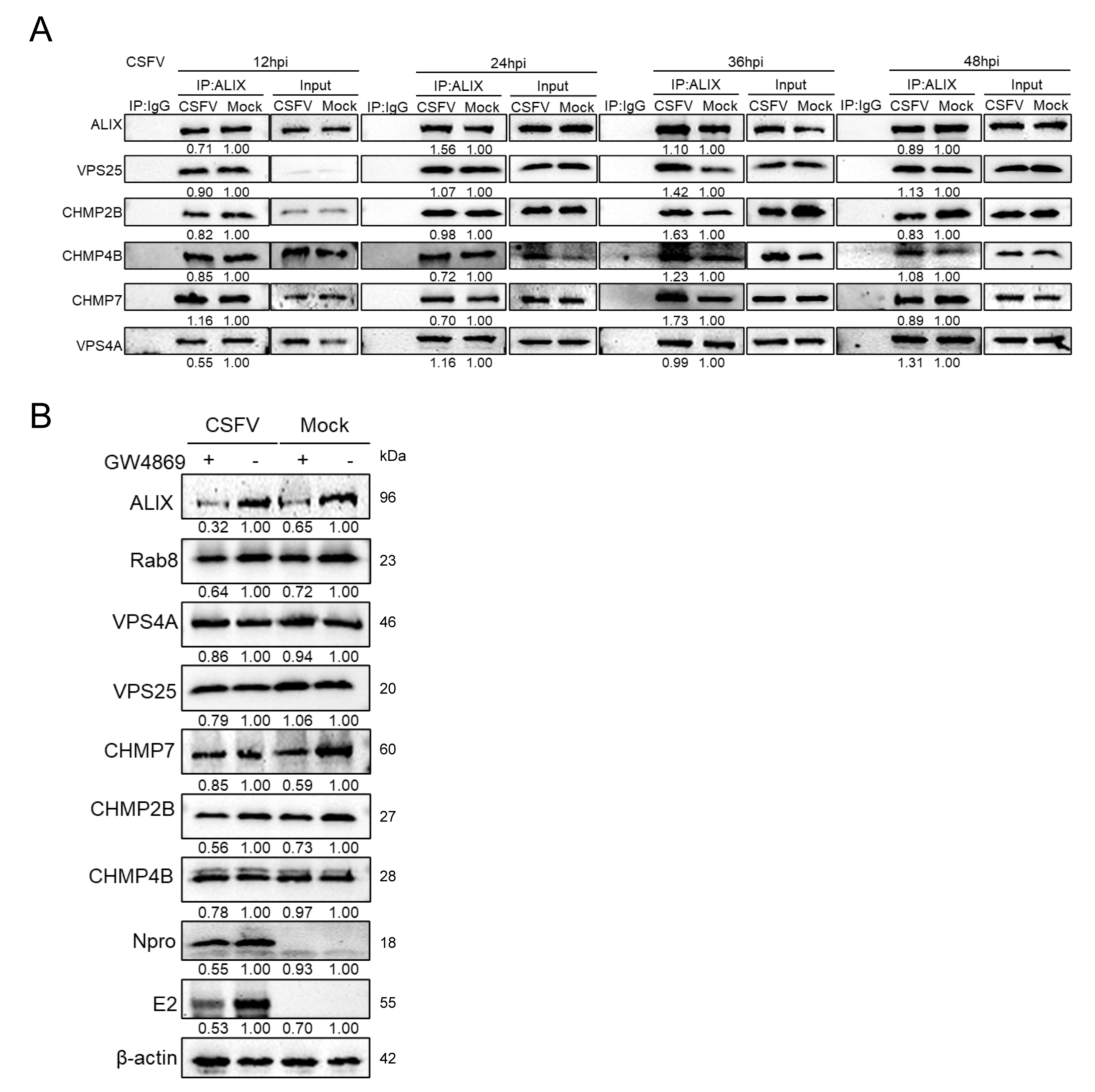
**

**S4 Fig. ESCRT-Ⅲ co-localizes with Flag-E2 upon CSFV infection**.

Cells transfected with pFlag-E2 were infected with CSFV (MOI=0.5) for 36 h, then fixed with 4% PFA and probed with rabbit anti-CHMP2B/CHMP4B/CHMP7 antibodies (green) and mouse anti-Flag antibody (red). Nuclei were stained with DAPI and observed by confocal microscopy. Scale bars = 10 µm. Data are presented as the mean ± SD of data from three independent experiments. **p < 0.05, **p < 0.01*.

**
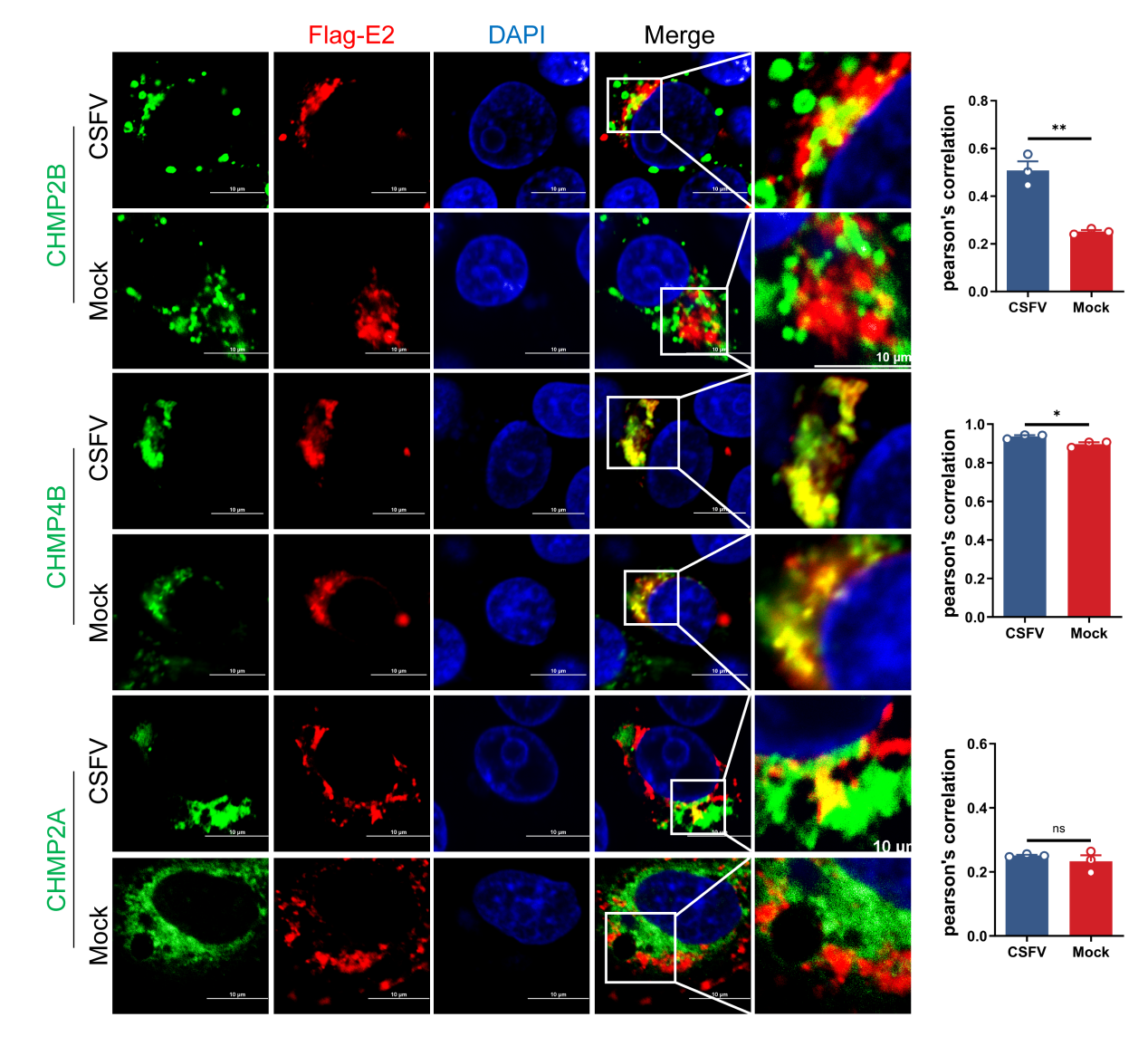
**

**S5 Fig. ESCRT-Ⅲ associated subunits are located in Golgi apparatus.**

PK-15 cells infected with CSFV (MOI=0.5) for 36 h were fixed and subjected to confocal microscopy using rabbit anti-CHMP2B/CHMP4B/CHMP7 antibody (green), [Golgi-Tracker](https://www.beyotime.com/product/C1043.htm) Red (red). Nuclei were stained with DAPI and observed by confocal microscopy. Scale bars = 10 µm. Data are presented as the mean ± SD of data from three independent experiments. ***p < 0.01, ***p < 0.001*.

**
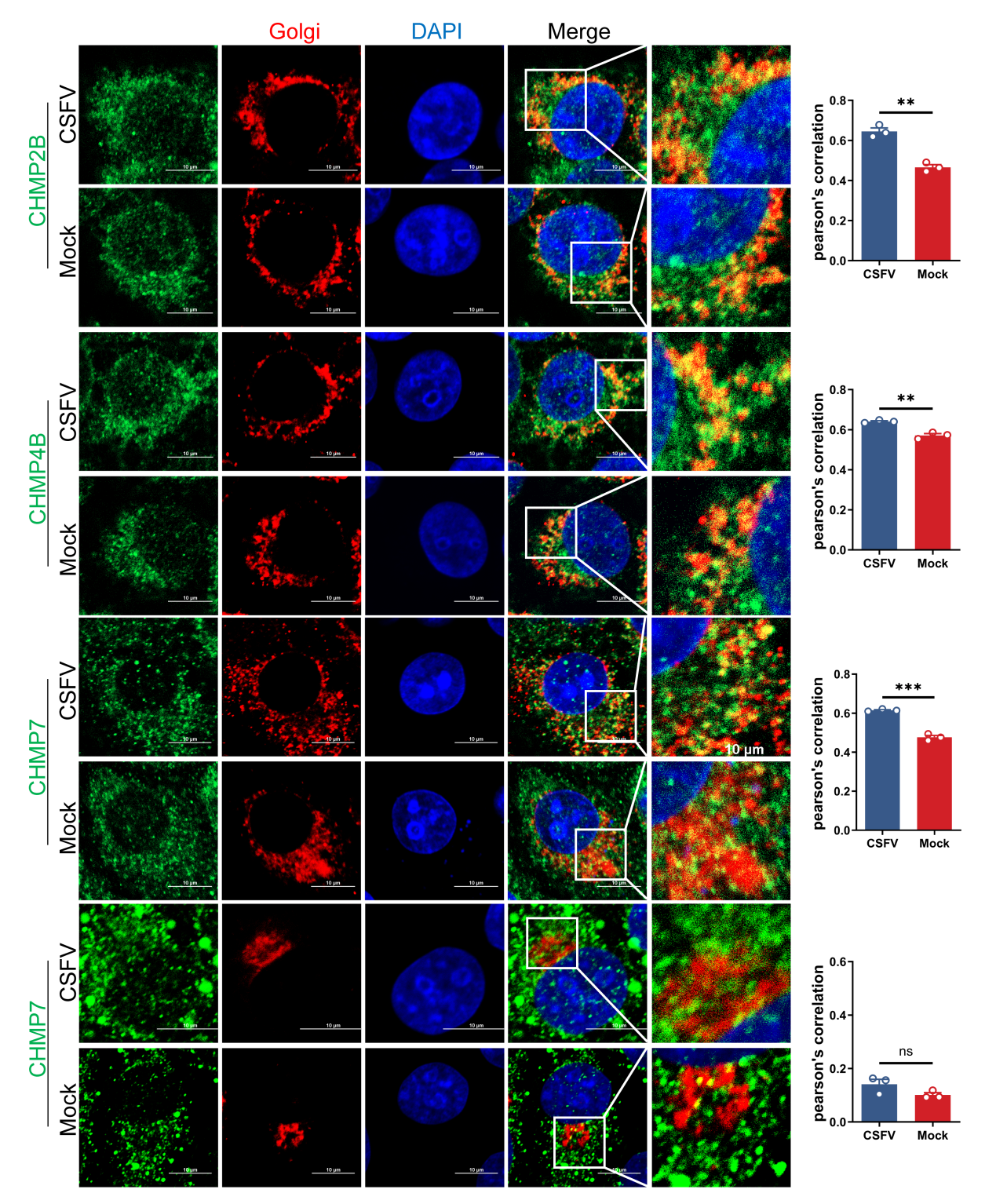
**

**S6 Fig. Rab8 is located in Golgi apparatus.**

1. Cells transfected with siALIX were infected with CSFV (MOI=0.5) for 36 h, then fixed and subjected to confocal microscopy using mouse anti-E2 antibody (green), rabbit anti-Rab9 or anti-Rab11 antibody (red). Nuclei were stained with DAPI. Scale bars = 10 µm. (B) PK-15 cells infected with CSFV (MOI=0.5) for 36 h were fixed and subjected to confocal microscopy using rabbit anti-Rab8 antibody (green), Golgi-Tracker (red) or ER-Tracker Red(red). Nuclei were stained with DAPI. Scale bars = 10 µm. Data are presented as the mean ± SD of data from three independent experiments. ***p < 0.01*.


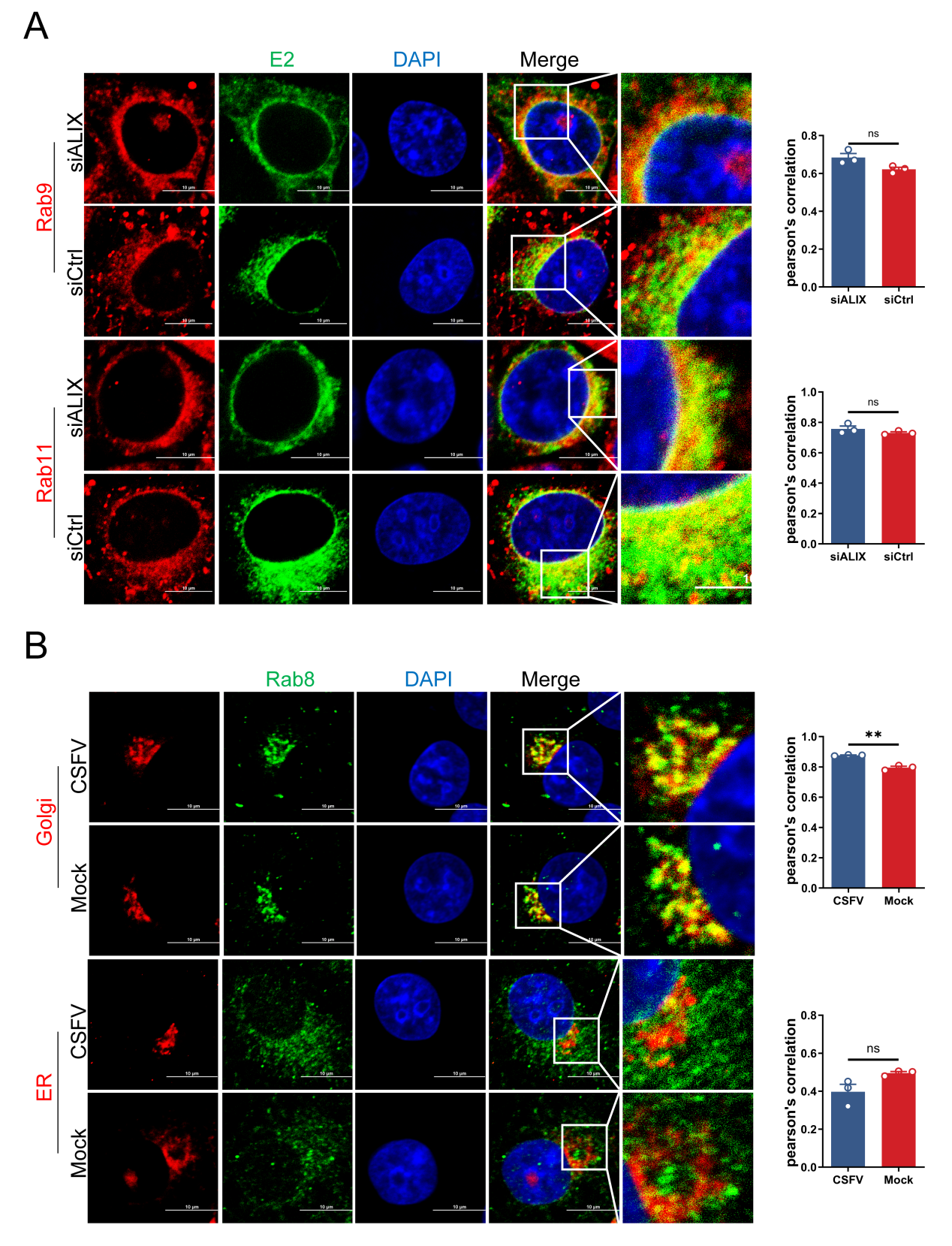


**S7 Fig. Expression of Rabs and Kinesins at various time points during CSFV infection.**

(A and B) PK-15 cells were infected CSFV (MOI=0.5) for 12, 24 and 36 h, and whole-cell lysates were collected and subjected to Western blotting using rabbit anti-Rab9, anti-Rab11, anti-Rab8, anti-Rab12, anti-Kif3A, anti-Kif4A, anti-Kif5A, anti-Kif5B, and anti-Npro antibodies, along with β-actin as a loading control.

**
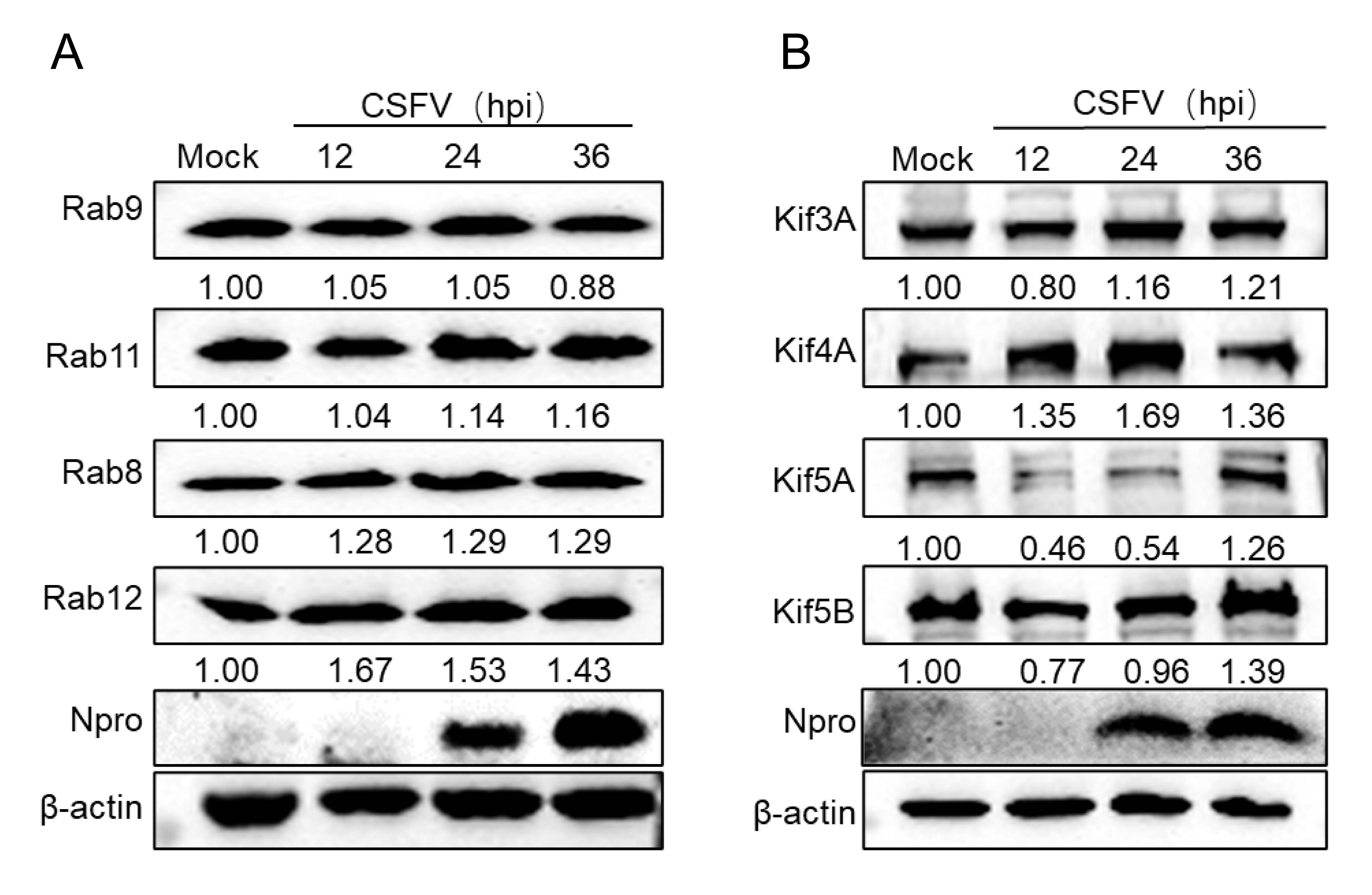
**

**S8 Fig. CHMP4B recruits Kif4A to assist in viral budding.**

1. PK-15 cells infected with CSFV (MOI=0.5) for 36 h were fixed and subjected to confocal microscopy using rabbit anti-Kif3A/Kif4A/Kif5A/Kif5B antibodies (green), mouse anti-CHMP4B antibody (red). Nuclei were stained with DAPI and observed by confocal microscopy. Scale bars = 10 µm. (B) PK-15 cells infected with CSFV (MOI=0.5) for 36 h were harvested and subjected to immunoprecipitation using rabbit anti-CHMP4B antibody. Whole-cell lysates were harvested and subjected to Western blotting using rabbit anti-Kif3A/Kif4A/Kif5A/Kif5B antibodies, rabbit anti-Tubulin antibody, mouse anti-Vimentin antibody. Data are presented as the mean ± SD of data from three independent experiments. ***p < 0.01, ***p < 0.001*.


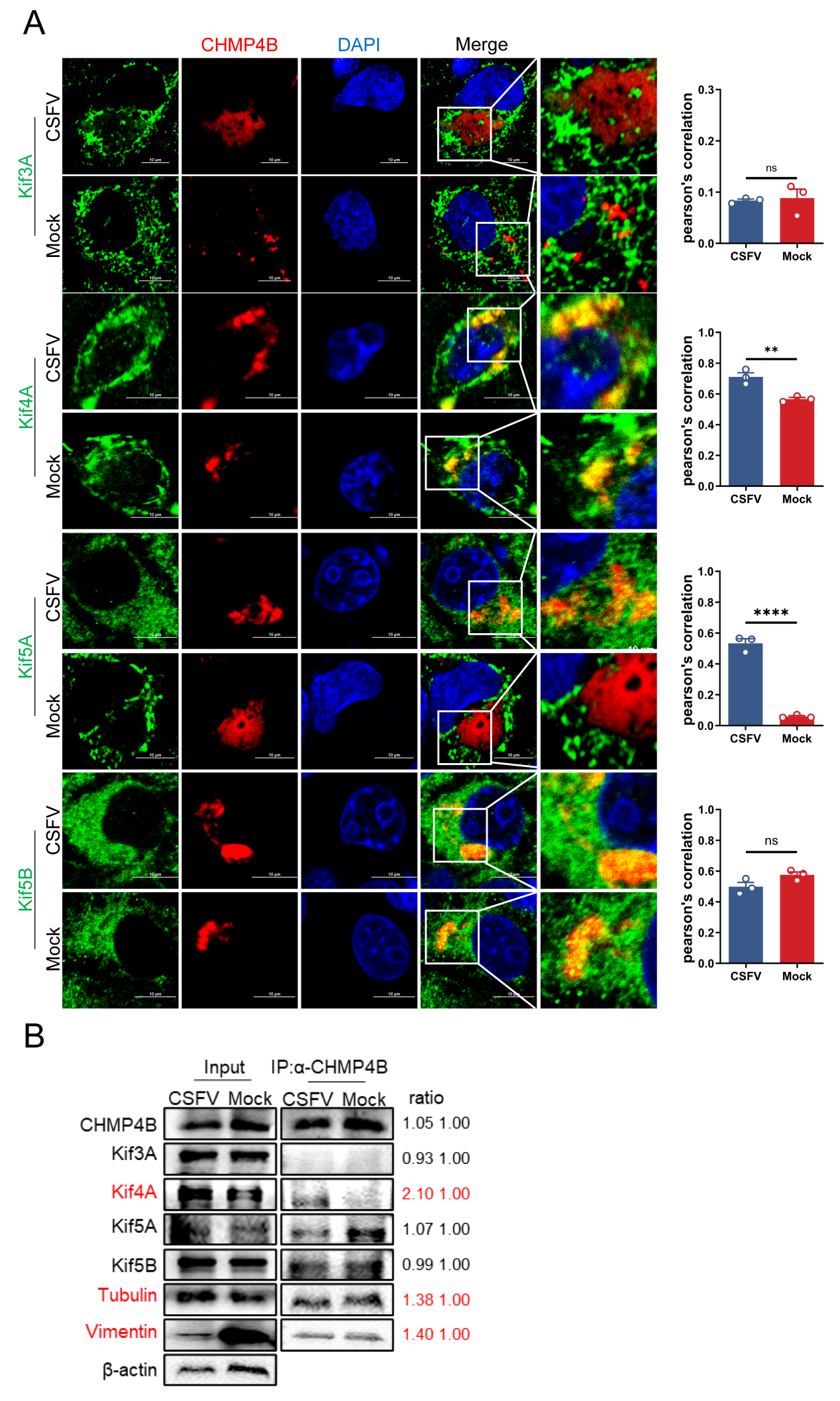

Supplement: Supplemental figures — Figs. S1–S8. [file mbio.02618-24-s0001.docx]
