# Supplementary material for: Classical swine fever virus recruits ALIX and ESCRT-III to facilitate viral budding
Source: mBio. 2025 Feb 25;16(4):e02618-24. doi: 10.1128/mbio.02618-24 (PMC11980558; doi:10.1128/mbio.02618-24)
Supplement: Supplemental tables — Tables S1 to S3. [file mbio.02618-24-s0002.docx]

**S1 Table 1. CSFV and BDV reference strains used in this study.**

| **Strain** | **Accession Number** |
| --- | --- |
| CSFV Shimen | AF092448 |
| CSFV Guizhou | KJ661548 |
| CSFV HCLV | GQ166162 |
| CSFV JL1 | EU497410 |
| CSFV TW/05 | GQ396706 |
| BDV Italy-58987 | MG649392 |
| BDV BD35-15 | MF102262 |
| BDV Frijters | MZ664275 |
| BDV R4785 | MF102260 |
| BDV R9336/11 | MF102261 |
| BDV 466 | AY163657 |

**S2 Table. Primers and siRNA duplexes used in this study.**

| **Primer** | **Sequence (5'-3'**） | **Accession Number** | **Use** |
| --- | --- | --- | --- |
| siCtrl | UUCUCCGAACGUGUCACGUTT | AJ312193 | siCtrl interference RNA |
| ALIX | GCCGCUGGUGAAGUUCAUCTT | XP020927281 | ALIX interference RNA |
| CHMP2B | UCGAGCAGCUUUAGAGAAATT | XM013990176 | CHM2B interference RNA |
| CHMP4B | AGAAAGAAGAGGAGGACGATT | XM003134402 | CHMP4B interference RNA |
| CHMP7 | GGGAGAAGAUUGUGAAGUUTT | XM021074113 | CHMP7 interference RNA |
| Rab8 | CCAGAGACAUCAAAGCAAA | NM213961 | Rab8 interference RNA |
| Kif4A | GGUGGUUGGUACUGAUAAA | XM005657819 | Kif4A interference RNA |
| CSFV-F | CCTGAGGACCAAACACATGTTG | AF092448 | RT-qPCR for detection of CSFV |
| CSFV-R | TGGTGGAAGTTGGTTGTGTCTG |  |  |
| ALIX-F | GCCTTGAGTCGAGAGCCTAC | XP020927281 | RT-qPCR for detection of ALIX |
| ALIX-R | GTGGTACTCAGCATTGGCCT |  |  |
| CHMP2B-F | AAGAAAACCGTGGATGGGCA | XM013990176 | RT-qPCR for detection of CHMP2B |
| CHMP2B-R | TGTGTGCCTCGTAACTCTCG |  |  |
| CHMP4B-F | CCAGAGACTGCGGGACACAG | XM003134402 | RT-qPCR for detection of CHMP4B |
| CHMP4B-R | CAGCTGCTTTTCATACCGCT |  |  |
| CHMP7-F | GGACACGCGGACTTATTTCA | XM021074113 | RT-qPCR for detection of CHMP7 |
| CHMP7-R | CTTTTGAAGGCGGAGAACAG |  |  |
| Rab8-F | CGTTCACCCCACATGAACTG | NM213961 | RT-qPCR for detection of Rab8 |
| Rab8-R | TTCCCTTTTAGTTTTCCAAG |  |  |
| Kif4A-F | CTGTAGCACCACTCATAAAA | XM005657819 | RT-qPCR for detection of Kif4A |
| Kif4A-R | AAGTATGATACTTTCAGAGT |  |  |
| ACTIN-F | GTGATCTCCTTCTGCATCCTGTC | AJ312193 | RT-qPCR for detection of ACTIN |
| ACTIN-R | CTCCATCATGAAGTGCGACGT |  |  |

**S3 Table. Antibodies used in this study.**

| **Antibody** | **Name** | **Supplier** | **Catalog no.** |
| --- | --- | --- | --- |
| β-Actin | [β-Actin Rabbit mAb (High Dilution)](https://abclonal.com.cn/catalog/AC026) | Abclonal | AC026 |
| Kif3A | KIF3A Rabbit pAb | Abclonal | A6639 |
| Kif4A | KIF4A Rabbit pAb | Abclonal | A10193 |
| Kif5A | KIF5A Rabbit pAb | Abclonal | A3303 |
| Kif5B | KIF5B Rabbit pAb | Abclonal | A15284 |
| TGN46 | [TGN46/TGOLN2 Rabbit mAb](https://abclonal.com.cn/catalog/A19618) | Abclonal | A19618 |
| STX4 | [Syntaxin 4 Rabbit mAb](https://abclonal.com.cn/catalog/A5996) | Abclonal | A5996 |
| VPS4A | [VPS4A Rabbit pAb](https://abclonal.com.cn/catalog/A7096) | Abclonal | A7096 |
| Rab8 | [RAB8A Rabbit mAb](https://abclonal.com.cn/catalog/A20976) | Abclonal | A20976 |
| Tubulin | [β-Tubulin Rabbit pAb](https://abclonal.com.cn/catalog/AC015) | Abclonal | AC015 |
| ALIX | ALIX Polyclonal antibody | Proteintech | 12422-1-AP |
| GFP | GFP tag polyclonal Ab | Proteintech | 50430-2-AP |
| CHMP7 | CHMP7 Polyclonal antibody | Proteintech | 4302 |
| VPS25 | VPS25 Polyclonal antibody | Proteintech | 15669-1-AP |
| Tsg101 | TSG101 Polyclonal antibody | Proteintech | 28283-1-AP |
| CHMP1B | CHMP1B Polyclonal antibody | Proteintech | 14639-1-AP |
| Rab12 | RAB12 Polyclonal antibody | Proteintech | 18843-1-AP |
| GM130 | GOLGA2/GM130 Polyclonal antibodies | Proteintech | 11308-1-AP |
| Calnexin | Calnexin Monoclonal antibody | Proteintech | 66903-1-Ig |
| CD63 | CD63 Monoclonal antibody | Proteintech | 67605-1-Ig |
| ALIX | ALIX Antibody (1H12) | Santa Cruz | Sc-53540 |
| CHMP1A | CHMP1A Antibody (B-5) | Santa Cruz | sc-271617 |
| CHMP5 | CHMP5 Antibody (F-7) | Santa Cruz | sc-374338 |
| CHMP6 | CHMP6 Antibody (B-3) | Santa Cruz | sc-398963 |
| HA | Anti-HA Antibody Mouse MAb | Sigma-Aldrich | H3663 |
| Flag | Anti-Flag M2 antibody | Sigma-Aldrich | F1804 |
| Flag-beads | Anti-flag ® M2 magnetic beads | Sigma-Aldrich | M8823 |
| Flag-beads | Anti-Flag Nanobody IP kit (Magarose) | AlpalifeBio | KTSM1361 |
| CHMP2B | CHMP2B (D4G3K) Rabbit mAb | Cell Signaling Technology | 76173 |
| CHMP4B | CHMP4B Antibody | Cell Signaling Technology | 42466 |
| HRS | HRS (D7T5N) Rabbit mAb | Cell Signaling Technology | 15087 |
| Rab8 | Rab8A (D22D8) XP® Rabbit mAb | Cell Signaling Technology | 6975 |
| Rab9 | Rab9A (D52G8) XP® Rabbit mAb | Cell Signaling Technology | 5118 |
| Rab11 | Rab11 (D4F5) XP® Rabbit mAb | Cell Signaling Technology | 5589 |
| Vimentin | Vimentin mouse monoclonal Antibody | Affinity | BF8006 |
